# Prevalence and trends of *Chlamydia trachomatis* infection in female sex workers and men who have sex with men in China: a systematic review and meta-analysis

**DOI:** 10.1186/s12889-024-18804-3

**Published:** 2024-06-12

**Authors:** Hui Jian, Wen-Jie Lu, Ze-Wei Chen, Shi-Qing Liang, Xiao-Li Yue, Jing Li, Jia-Hui Zhang, Xiang-Dong Gong

**Affiliations:** 1 Hospital for Skin Diseases, Institute of Dermatology, Chinese Academy of Medical Sciences and Peking Union Medical College, Nanjing, China; 2 School of Public Health, Nanjing Medical University, Nanjing, China; 3 Department of STD Epidemiology, National Center for STD Control, Nanjing, China

**Keywords:** *Chlamydia trachomatis*, Female sex workers, Men who have sex with men, Prevalence, Meta-analysis, China

## Abstract

**Introduction:**

*Chlamydia trachomatis* infection can cause a significant disease burden in high-risk populations. This study aimed to assess the overall prevalence of *C. trachomatis* infection, and determine the long-term trends and geographic distribution of this infection among female sex workers (FSWs) and men who have sex with men (MSM) in China.

**Methods:**

The PubMed, Web of Science, CNKI, Wanfang Data and VIP databases were searched from 1 January 1990 through 30 April 2023. Publications in which *C. trachomatis* infection was detected using nucleic acid amplification tests (NAATs) were included. The *Q* test and *I*^2^ statistics were used to assess the heterogeneity between studies. A random-effect model was used to estimate the pooled prevalence of *C. trachomatis* infection. Subgroup, meta-regression, and sensitivity analyses were performed to explore the sources of heterogeneity. Publication bias was evaluated using Egger’s test. Trend analysis of the prevalence was performed using the Jonckheere-Terpstra trend test method.

**Results:**

Sixty-one studies were eligible for inclusion (including 38 for FSWs and 23 for MSM). The pooled prevalence of *C. trachomatis* infection was 19.5% (95% CI: 16.4, 23.0) among FSWs and 12.7% (95% CI: 9.2, 17.7) in the rectum, 6.4% (95% CI: 5.3, 7.8) in the urethra and 1.3% (95% CI: 0.8, 2.1) in the oropharynx from MSM in China. The subgroup analyses showed that the sample size, study period, study region, specimen collection type, molecular diagnosis method, and recruitment site could explain some heterogeneity among studies of FSWs, and the publication language, study period, study region, molecular diagnosis method, and specimen collection anatomical site could explain some heterogeneity among studies of MSM. From 1998 to 2004, 2005 to 2009, 2010 to 2015, and 2016 to 2021, the pooled prevalence of *C. trachomatis* infection among FSWs were 30.3%, 19.9%, 21.4%, and 11.3%, respectively. For MSM, the pooled prevalence from 2003 to 2009, 2010 to 2015, and 2016 to 2022 were 7.8%, 4.7%, and 6.5%, respectively. However, no overall decline in the prevalence of *C. trachomatis* infection was observed among FSWs (z = -1.51, *P* = 0.13) or MSM (z = -0.71, *P* = 0.48) in China.

**Conclusions:**

The prevalence of *C. trachomatis* infection was high in these two high-risk populations in China. The findings of this study provide evidence for the formulation of effective surveillance and screening strategies for the prevention and control of *C. trachomatis* infection among these two specific populations.

**Supplementary Information:**

The online version contains supplementary material available at 10.1186/s12889-024-18804-3.

## Introduction

Genital infection caused by the bacterium *Chlamydia trachomatis* is currently the most pervasive and commonly diagnosed bacterial sexually transmitted disease (STD) worldwide [1]. *C. trachomatis* infection is asymptomatic in more than 70% of infected women and approximately 50% of infected men and, as a consequence, often remains undiagnosed and untreated [2]. If left untreated or treated inadequately, this infection can result in severe complications and long-term adverse outcomes in the reproductive system, including pelvic inflammatory disease, ectopic pregnancy, and infertility in women and epididymitis in men [3]. Early detection and treatment are needed to reduce the burden of *C. trachomatis* infection and associated sequelae.

The World Health Organization (WHO) estimated that there were approximately 128 million new cases of *C. trachomatis* infection among adults worldwide in 2020 [4]. In 2019, 50,874 new cases of genital *C. trachomatis* infection were reported from 105 national STD surveillance sites in China, which corresponded to 55.32 incident cases per 100,000 population [5] and constituted a 70.32% increase from 2008 (32.48 incident cases per 100,000 population) [6]. However, national STD surveillance sites in China provide enhanced case reporting within sampling districts and not prevalence surveys in high-risk populations; therefore, specific information on FSWs and MSM is not collected at these surveillance sites [7].

FSWs and MSM are at high risk of contracting STDs and have significantly greater prevalence rates of *C. trachomatis* infection than the general population [8, 9]. Several studies have investigated the prevalence of *C. trachomatis* infection among FSWs and MSM in China. For instance, a meta-analysis reported a pooled prevalence of 16.4% (2006–2015) for genital *C. trachomatis* infection among FSWs in China [10]. Another study showed a prevalence of 4.3% (urethral) and 16.5% (rectal) for *C. trachomatis* infection among MSM (2000–2013) [11]. However, the studies included in these two meta-analyses used nucleic acid amplification tests (NAATs), enzyme-linked immunosorbent assays or cell culture methods for *C. trachomatis* detection. NAATs are much more sensitive than the other two methods and are recommended as the optimal methods for diagnosing genital and extragenital infections caused by *C. trachomatis* in symptomatic or asymptomatic individuals [12]. In addition, these two meta-analyses were published before 2016 and did not analyse the temporal trends and geographic distribution of *C. trachomatis* infection among FSWs and MSM.

Therefore, we performed this systematic review and meta-analysis by including only studies that used NAATs to update national and regional prevalence estimates for *C. trachomatis* infection and long-term trend changes in this infection among two high-risk populations. This study aimed to provide convincing information for a comprehensive assessment of the disease burden of *C. trachomatis* infection and to enhance surveillance of the prevalence of *C. trachomatis* infection among these two at-risk populations across the country.

## Methods

This meta-analysis conformed to the Preferred Reporting Items for Systematic Reviews and Meta-Analyses (PRISMA) guidelines [13] (Supplementary Table A) and was preregistered to the International Prospective Register of Systematic Reviews (PROSPERO) with the registration number CRD42022375841 (URL: https://www.crd.york.ac.uk/prospero/display_record.php?RecordID=375841).

### Search strategy

We performed a thorough literature search for articles published between 1 January 1990 and 30 April 2023 in several databases, including PubMed, Web of Science, the Chinese National Knowledge Infrastructure (CNKI), Wanfang Data and the Chinese Scientific Journals Database (VIP). Additionally, we checked the reference lists of relevant articles for additional studies. The combinations of search terms included ‘*Chlamydia trachomatis’*, ‘genital infection’, ‘prevalence’, ‘female sex workers’, ‘FSWs’, ‘men who have sex with men’, ‘MSM’, ‘homosexual men’, ‘gay’, and ‘China’. The search language was limited to English and Chinese. The search strategies are detailed in Supplementary Table B.1. We included only studies conducted in mainland China. Studies conducted in Hong Kong, Macau or Taiwan were excluded.

### Inclusion and exclusion criteria

To satisfy the analysis requirements, studies needed to meet the following criteria: 1) the study participants were FSWs or MSM from China, as defined in Supplementary Box C; 2) the language of the article was English or Chinese; 3) the study was a cross-sectional survey or provided cross-sectional survey data with a valid sample size, such as baseline data for a cohort study; 4) the study reported the prevalence of *C. trachomatis* infection, including the numerator and denominator; and 5) *C. trachomatis* infection was detected using NAATs.

The following criteria were used to exclude studies: 1) studies whose participants and research methods did not meet the above criteria; 2) FSWs and MSM were infected with human immunodeficiency virus (HIV) or were injection drug users; 3) studies in which participants were seeking care only for sexually transmitted infections (STIs) or genital symptoms; 4) studies that did not describe laboratory testing methods or specimen collection types; and 5) studies with incomplete data that did not allow us to estimate the prevalence or determine the survey time. If multiple articles were based on the same population at the same study time, only the study that reported the most detailed data was included.

### Study selection and quality assessment

Authors HJ and WL screened the titles and abstracts of all records identified through electronic searches and made initial judgements about eligibility. Studies that did not meet the inclusion criteria or were irrelevant were excluded during this stage. If a study could not be definitively excluded based on its title and abstract, the full text was obtained for comprehensive screening. Review articles were removed after we conducted manual searches of their references. Dissertations and conference abstracts were included if they met the eligibility criteria.

Authors HJ and WL then independently reviewed the full texts of the eligible articles and assessed the quality of the included studies using the Agency for Healthcare Research and Quality (AHRQ) checklist tool [14]. This tool has 11 items, and each item on the checklist was scored as ‘1’ for ‘YES’, ‘0’ for ‘NO’ or ‘UNCLEAR’. The final quality assessment scores were as follows: low quality = 0–3 points; moderate quality = 4–7 points; and high quality = 8–11 points. The quality assessment results are summarized in Supplementary Table D.

### Data extraction

The review process involved extracting relevant data from eligible studies using a form developed by reviewers HJ and WL. To validate the accuracy of the extracted data, reviewers HJ, WL, CZ and LS examined all of the studies. The following information was collected: the first author, publication title, publication year, publication language, study period, study region, study population, study design, sample size, number of positive cases of *C. trachomatis* infection, laboratory testing method, specimen collection type, and molecular diagnosis method; the recruitment site was exclusively collected for FSWs, and the specimen collection anatomical site was exclusively collected for MSM. The details of the extracted variables are presented in Supplementary Box B.

### Statistical analysis

The meta-analysis was conducted using the R meta package (version 6.0–0) of the R software (version 4.1.0). To stabilize the variance and improve statistical properties, the raw proportions were transformed to more closely approximate a normal distribution. The prevalence of *C. trachomatis* infection in the FSW population was transformed using logit transformation, while the prevalence among MSM was transformed using the Freeman-Tukey double arcsine transformation [15]. We assessed heterogeneity between studies by using the *Q* test, with a *p* value of less than 0.10 considered to indicate statistically significant heterogeneity, and the *I*^2^ statistic, with values of 25%, 50%, and 75% indicating low, medium, and high heterogeneity, respectively [16]. We performed a meta-analysis using a random-effects model to estimate the pooled prevalence of *C. trachomatis* infection and its 95% confidence interval (CI) because of substantial heterogeneity across studies. The Jonckheere-Terpstra trend test method [17] was employed to assess the temporal trend of the prevalence. Bubble charts were drawn to visualize the temporal trends in prevalence. The ArcGIS 10.5 software was used to plot the distribution of prevalence in different regions.

To explore the associations of the prevalence of *C. trachomatis* infection with different variables, subgroup analyses were performed based on the sample size, publication language, study period, study region (six geographical regions of China), specimen collection type, molecular diagnosis method, recruitment site (for FSWs), and specimen collection anatomical site (for MSM). We used the* Q* test based on the fixed-effects model to analyse differences between subgroups [18]. Specifically, when a study spanned two periods, it was allocated into subgroups for each period. Similarly, when a study conducted surveys across different regions, these surveys were assigned to regional subgroups. We also conducted random-effects multivariable meta-regression analyses to examine possible sources of heterogeneity with the following covariates: sample size, publication language, study period, study region, specimen collection type, molecular diagnosis method, recruitment site (for FSWs) and study quality. If a study variable could be divided into two or more subgroups, for example, across regions or periods, the study was excluded from the meta-regression analysis.

We used Egger’s test and visual inspection of a funnel plot to assess the potential for publication bias, with a *p* value < 0.1 considered to indicate statistical significance [19]. Sensitivity analysis was conducted by omitting studies one by one to assess each study's influence on the overall estimate.

## Results

### Study selection

**Fig. 1:**
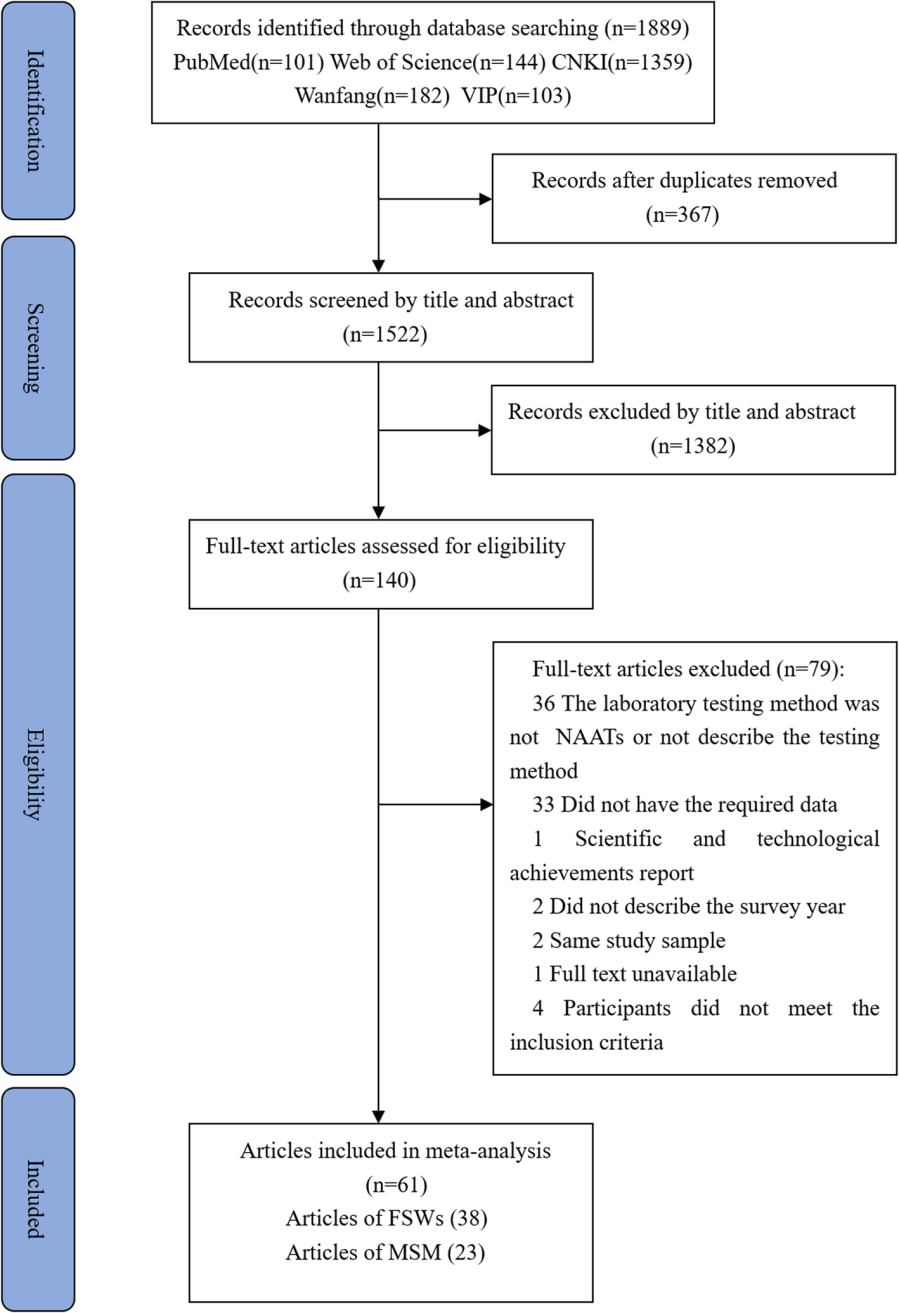
Flow diagram of the study selection process for the meta-analysis

A total of 1,889 articles were retrieved and screened to identify eligible publications (Fig. 1). After removing 367 duplicates, we screened the remaining 1,522 studies based on a review of their titles and abstracts. We then evaluated 140 full-text articles for eligibility, of which 79 were excluded (as described in Supplementary Table B.2). After a full-text review, we finally included 61 articles (38 on FSWs and 23 on MSM) in this meta-analysis. The publication years ranged from 2001 to 2023, and the study period for FSWs ranged from 1998 to 2021, while that for MSM ranged from 2003 to 2022.

### Study characteristics

The characteristics of the included studies are presented in Supplementary Tables C.1 and C.2. Among the studies on FSWs, 24 (63.2%) were published in Chinese, and 14 (36.8%) were published in English, and the studies were conducted in five regions of China with 29,022 participants. Among the studies on MSM, 15 (65.2%) were published in Chinese, and 8 (34.8%) were published in English, with a total of 8,556 participants, and no studies were found in northern and northwestern China. There was no low-quality literature. In brief, 41 studies (67.2%) had high precision, and 20 studies (32.8%) had moderate precision based on the AHRQ cross-sectional quality evaluation (Supplementary Table D).

### Pooled prevalence of *C. trachomatis* infection among FSWs and MSM

Although our original intention was to analyse the prevalence of *C. trachomatis* infection from 1990 to 2023, only studies on FSWs published during 2001–2023 (study period: 1998–2021) and studies on MSM published during 2006–2023 (study period: 2003–2022) were retrieved. According to the 38 articles on FSWs included in our study, the pooled prevalence of *C. trachomatis* infection among FSWs in China from 1998 to 2021 was 19.5% (95% CI: 16.4, 23.0), with a range from 4.3% (95% CI: 3.6, 5.0) to 58.6% (95% CI: 54.2, 63.0). According to the 23 articles on MSM included in our study, the pooled prevalence of *C. trachomatis* infection among MSM in China from 2003 to 2022 was 6.4% (95% CI: 5.3, 7.8) in the urethra, with a range from 3.0% (95% CI: 1.1, 6.4) to 16.2% (95% CI: 12.7, 20.1). The *Q* test indicated significant heterogeneity between studies for both FSWs (*I*^2^ = 97.0%,* P* < 0.01) and MSM (*I*^2^ = 87.0%,* P* < 0.01). Therefore, a random-effects model was used to calculate the pooled prevalence of *C. trachomatis* infection and the 95% CI.

### Subgroup analyses

As presented in Table 1, we conducted subgroup analyses based on the sample size, publication language, study period, study region, specimen collection type and molecular diagnosis method for FSWs and MSM. We also performed a subgroup analysis for FSWs by recruitment site and for MSM by anatomical specimen collection site. All subgroups showed statistically significant differences, except for the publication language among studies on FSWs and the sample size and specimen collection type among studies on MSM.

**Table 1:** Subgroup analysis of the factors associated with the prevalence of *C. trachomatis* infection among FSWs and MSM

Category	Number of studies	Number of participants	Prevalence, % (95% CI)	Heterogeneity		*χ*^*2*^	*p* value
				*I*^2^, %	*p* value		
**FSWs**	38	29,022	19.5(16.4–23.0)	97.0	< 0.01	—	—
**Sample size**							
< 572	19	5418	22.6(17.4–28.9)	97.0	< 0.01	178.13	< 0.01
≥ 572	19	23,604	16.9(13.8–20.4)	98.0	< 0.01		
**Publication language**							
Chinese	24	13,248	19.5(16.3–23.0)	94.0	< 0.01	3.38	0.07
English	14	15,774	19.7(13.8–27.2)	99.0	< 0.01		
**Study period**							
1998–2004	6	2668	30.3(20.5–42.4)	98.0	< 0.01	796.37	< 0.01
2005–2009	22	15,292	19.9(16.7–23.7)	93.0	< 0.01		
2010–2015	7	4020	21.4(16.9–26.7)	91.0	< 0.01		
2016–2021	7	8760	11.3(7.6–16.6)	97.0	< 0.01		
**Study region**							
Central-south	15	6708	20.4(17.1–24.2)	91.0	< 0.01	331.23	< 0.01
East	10	10,198	14.8(10.2–21.1)	97.0	< 0.01		
Southwest	16	11,067	21.7(16.2–28.4)	98.0	< 0.01		
Northwest	2	701	12.7(7.4–21.0)	92.0	< 0.01		
Northeast	1	348	6.9(4.5–10.1)	Not estimable	Not estimable		
**Specimen collection types**							
Cervical swab	26	18,320	20.6(17.8–23.7)	94.0	< 0.01		
Vaginal swab	2	928	31.8(9.0–68.7)	99.0	< 0.01	609.71	< 0.01
Urine	8	8367	14.8(8.8–24.0)	98.0	< 0.01		
**Molecular diagnosis methods**							
DNA	5	5078	14.9(12.2–18.1)	85.0	< 0.01	168.07	< 0.01
RNA	2	3682	4.5(3.9–5.2)	72.0	0.06		
**Recruitment sites**							
Entertainment venues	32	27,424	18.1(15.5–21.1)	97.0	< 0.01	298.97	< 0.01
DRI and others	6	1598	28.6(16.0–45.7)	98.0	< 0.01		
**MSM**	23	8556	6.4(5.3–7.8)	87.0	< 0.01	—	—
**Sample size**							
< 293	12	2074	7.0(5.7–8.4)	26.0	0.19	1.57	0.21
≥ 293	11	6482	6.0(4.0–8.4)	92.0	< 0.01		
**Publication language**							
Chinese	15	5653	7.2(5.5–9.2)	87.0	< 0.01	24.47	< 0.01
English	8	2903	4.9(3.8–6.2)	45.0	0.08		
**Study period**							
2003–2009	8	1935	7.8(5.6–10.4)	78.0	< 0.01		
2010–2015	6	1974	4.7(3.8–5.7)	13.0	0.33	21.34	< 0.01
2016–2022	12	5539	6.5(4.7–8.5)	88.0	< 0.01		
**Study region**							
Central-south	9	2143	6.9(4.5–9.7)	84.0	< 0.01	19.06	< 0.01
East	10	3330	5.2(4.2–6.3)	41.0	0.09		
Southwest	5	2906	6.7(3.5–10.9)	93.0	< 0.01		
Northeast	1	177	7.3(4.0–12.2)	Not estimable	Not estimable		
**Specimen collection types**							
Urethral swab	6	1051	5.9(4.1–7.9)	33.0	0.19	0.95	0.33
Urine	17	7505	6.6(5.0–8.4)	88.0	< 0.01		
**Molecular diagnosis methods**							
DNA	11	4452	6.7(5.0–9.0)	87.0	< 0.01	19.69	< 0.01
RNA	1	1087	4.2(3.1–5.6)	Not estimable	Not estimable		
**Specimen collection anatomical sites**							
Urethra	23	8556	6.4(5.3–7.8)	87.0	< 0.01		
Rectum	11	3055	12.7(9.2–17.7)	92.0	< 0.01	204.60	< 0.01
Oropharynx	6	2565	1.3(0.8–2.1)	41.0	0.13		

Prior to 2016, studies on these two high-risk populations detected DNA from *C. trachomatis*; therefore, in the subgroup analyses by molecular diagnostic methods, studies on FSWs were from 2016 to 2021, and those on MSM were from 2016 to 2022

*DRI* Detention and reeducation institutes, *CI* Confidence interval

For FSWs, the subgroup analysis by study period indicated that the pooled prevalence of *C. trachomatis* infection was the lowest in studies conducted from 2016 to 2021 (11.3%, 95% CI: 7.6, 16.6) and the highest in those conducted from 1998 to 2004 (30.3%, 95% CI: 20.5, 42.4). According to the subgroup analysis by the study region, the pooled prevalence of *C. trachomatis* infection was the highest in southwestern China (21.7%, 95% CI: 16.2, 28.4) and the lowest in northeastern China (6.9%, 95% CI: 4.5, 10.1). According to the subgroup analysis by the specimen collection type, the difference among the groups was statistically significant (*P* < 0.01). The pooled prevalence of *C. trachomatis* infection in cervical swabs, vaginal swabs and urine was 20.6% (95% CI: 17.8, 23.7), 31.8% (95% CI: 9.0, 68.7), and 14.8% (95% CI: 8.8, 24.0), respectively. The subgroup analysis by the molecular diagnosis method revealed that studies based on *C. trachomatis* DNA detection reported a greater prevalence of infection than did studies based on *C. trachomatis* RNA detection from 2016 to 2021 (14.9% versus 4.5%, *P* < 0.01). FSWs from entertainment venues had a lower prevalence of *C. trachomatis* infection than did those from detention and reeducation institutes and other recruitment sites (18.1% versus 28.6%, *P* < 0.01).

For MSM, the subgroup analysis by the study period revealed that the pooled prevalence of *C. trachomatis* infection was the highest in studies conducted from 2003 to 2009 (7.8%, 95% CI: 5.6, 10.4) and the lowest in those conducted from 2010 to 2015 (4.7%, 95% CI: 3.8, 5.7). According to the subgroup analysis by the study region, northeastern China had the highest pooled prevalence of *C. trachomatis* infection at 7.3% (95% CI: 4.0, 12.2), followed by central-southern China at 6.9% (95% CI 4.5, 9.7). The subgroup analysis by the molecular diagnosis method revealed that studies based on *C. trachomatis* DNA detection reported a greater prevalence of infection than those based on *C. trachomatis* RNA detection during the period of 2016–2022 (6.7% versus 4.2%, *P* < 0.01). According to the subgroup analysis by the specimen collection anatomical site, the difference among the groups was statistically significant (*P* < 0.01). The pooled prevalence of *C. trachomatis* infection in the rectum, urethra and oropharynx was 12.7% (95% CI: 9.2, 17.7), 6.4% (95% CI: 5.3, 7.8), and 1.3% (95% CI: 0.8, 2.1), respectively.

### Meta-regression analysis

The results of our multivariable meta-regression analysis for FSWs indicated that the study period (2016–2021: coefficient = -1.11,* P* = 0.02), study region (east: coefficient = -0.65,* P* = 0.03) and specimen collection type (urine: coefficient = 0.68,* P* = 0.03) contributed to the highest heterogeneity across studies (Table 2).

**Table 2:** Raw coefficients from the multivariable meta-regression analysis

Predictor	Number of studies	Number of participants	Coefficient	*p* value
**FSWs**				
** Sample size**				
< 572	16	4505	Ref	Not applicable
≥ 572	15	19,154	-0.12	0.49
** Publication language**				
Chinese	19	10,525	Ref	Not applicable
English	12	13,134	0.17	0.34
** Study period**				
1998–2004	6	2668	Ref	Not applicable
2005–2009	17	11,967	0.03	0.94
2010–2015	2	1269	0.24	0.64
2016–2021	6	7755	-1.11	0.02
** Study region**				
Central-south	8	3808	Ref	Not applicable
East	8	9789	-0.65	0.03
Southwest	15	10,062	-0.03	0.91
** Specimen collection types**				
Cervical swab	21	14,364	Ref	Not applicable
Urine	8	8367	0.68	0.03
Vaginal swab	2	928	0.78	0.06
** Molecular diagnosis methods**				
DNA	29	19,977	Ref	Not applicable
RNA	2	3682	-0.79	0.08
** Recruitment sites**				
DRI and others	5	1500	Ref	Not applicable
Entertainment venues	26	22,159	-0.42	0.23
** Study quality**				
High	20	13,418	Ref	Not applicable
Moderate	11	10,241	0.04	0.86
**MSM**				
** Sample size**				
< 293	10	1492	Ref	Not applicable
≥ 293	9	5793	-0.01	0.83
** Publication language**				
Chinese	13	5071	Ref	Not applicable
English	6	2214	-0.04	0.47
** Study period**				
2003–2009	6	1353	Ref	Not applicable
2010–2015	3	1082	-0.11	0.07
2016–2022	10	4850	-0.05	0.43
** Study region**				
Central-south	5	1405	Ref	Not applicable
East	8	2797	-0.02	0.68
Northeast	1	177	0.07	0.57
Southwest	5	2906	-0.02	0.74
** Specimen collection types**				
Urethral swab	6	1051	Ref	Not applicable
Urine	13	6234	0.05	0.45
** Moleculardiagnosis methods**				
DNA	18	6198	Ref	Not applicable
RNA	1	1087	-0.06	0.58
** Study quality**				
High	16	5610	Ref	Not applicable
Moderate	3	1675	0.03	0.71

### Trends in the prevalence of *C. trachomatis* infection among FSWs and MSM in China over time

The meta-regression results indicated that for FSWs, there was a lower prevalence of *C. trachomatis* infection during the period of 2016–2021 than during the reference period (*P* = 0.02). By checking the original data for FSWs, we found that studies conducted during the periods of 1998–2004, 2005–2009, and 2010–2015 involved the detection of only *C. trachomatis* DNA; during 2016 to 2021, 5 studies used *C. trachomatis* DNA detection, and 2 studies used RNA detection. The prevalence of *C. trachomatis* infection was significantly different between these two molecular diagnostic methods, and studies reported that *C. trachomatis* RNA detection resulted in a lower prevalence (14.9% vs. 4.5%, *χ*^2^ = 168.07, *P* < 0.01). A similar situation was observed for MSM. During the periods of 2003–2009 and 2010–2015, all studies used *C. trachomatis* DNA detection. From 2016 to 2022, out of 12 studies, 11 used *C. trachomatis* DNA detection, and 1 study that used RNA detection showed a lower prevalence of *C. trachomatis* infection (6.7% vs. 4.2%, *χ*^2^ = 19.69, *P* < 0.01). Therefore, to avoid the impact of molecular diagnosis methods on trend analysis, we included only studies that were based on *C. trachomatis* DNA detection.

**Fig. 2:**
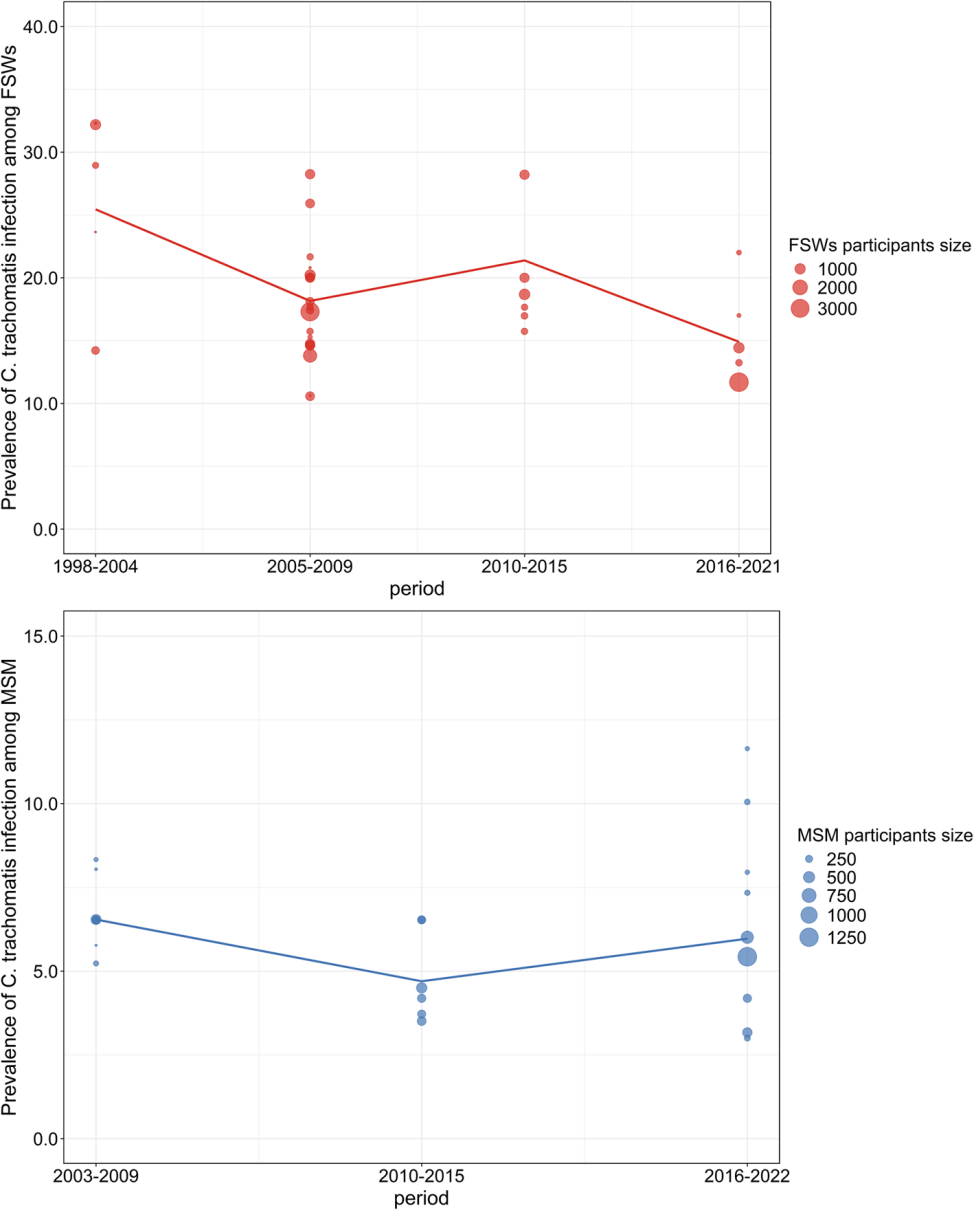
Time-trend bubble plots of the prevalence of *C. trachomatis* infection among FSWs and MSM in China. Note: Only studies that were based on *C. trachomatis* DNA detection were included, and only data on MSM urethral infections were included, while rectal and oropharyngeal data were not included

The studies of FSWs were conducted from 1998 to 2021 and were divided into four periods. The studies among MSM covered the years 2003–2022 and were divided into three periods. Two bubble plots were used to illustrate the trends in the prevalence of *C. trachomatis* infection over time among FSWs and MSM, as depicted in Fig. 2. After removing three outliers from the studies of FSWs and two outliers from the studies of MSM, the results of the Jonckheere-Terpstra trend test showed that there were no decreasing trends in the prevalence of *C. trachomatis* infection among both FSWs (z = -1.51, *P* = 0.13) and MSM (z = -0.71, *P* = 0.48).

### Distribution and comparison of the prevalence of *C. trachomatis* infection in the two high-risk populations in different geographical areas

**Fig. 3:**
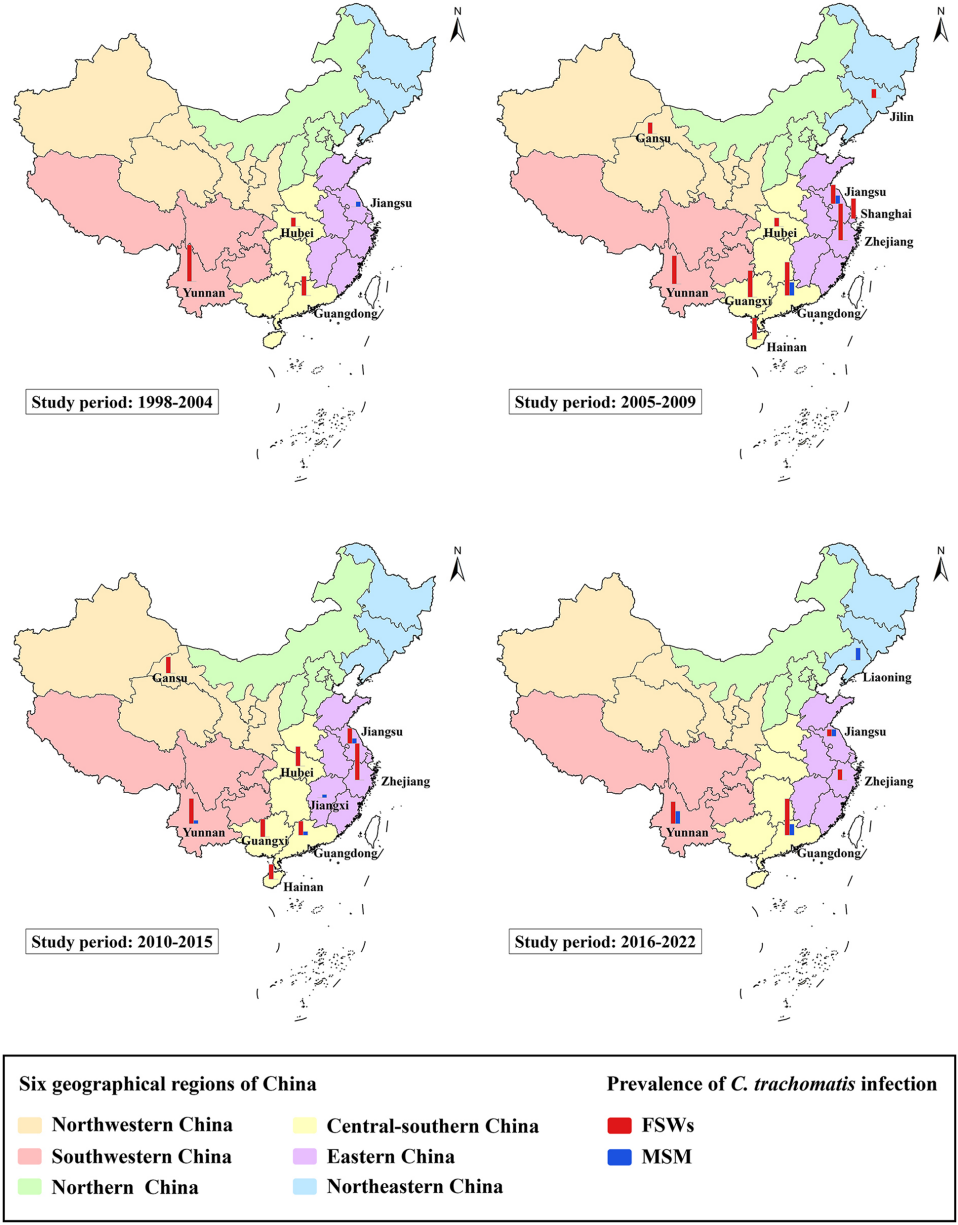
Prevalence of *C. trachomatis* infection among FSWs and MSM in different geographical areas of China

To visualize differences in the prevalence of *C. trachomatis* infection in different geographical areas of China among the two high-risk populations, we constructed bar charts of the prevalence of *C. trachomatis* infection among FSWs and MSM, as depicted in Fig. 3. Subgroup analysis revealed statistically significant differences in the prevalence of *C. trachomatis* infection across different periods in these two high-risk populations; therefore, we divided the study periods into four intervals. Overall, the prevalence of *C. trachomatis* infection among FSWs and MSM varied across provinces over time. The number of provinces that conducted surveys on FSWs initially increased and then decreased, while the number of provinces that conducted research on MSM steadily increased. Studies on these two groups were mainly conducted in the southeastern coastal and southwestern regions of China. The prevalence of *C. trachomatis* infection in these two populations was visually different between provinces. Three provinces conducted studies on the prevalence of *C. trachomatis* infection in both FSWs and MSM, and all showed a greater prevalence of *C. trachomatis* infection in FSWs than in MSM, including in provinces such as Yunnan and Guangdong.

### Sensitivity analysis and publication bias

According to the sensitivity analysis (Supplementary Table E), the pooled prevalence of *C. trachomatis* infection among FSWs and MSM was found to be robust and not significantly affected, indicating the reliability of the results. Additionally, the leave-one-out method was applied to assess the influence of individual studies on the pooled estimate, and the results showed that no single study had a disproportionate effect on the overall estimate (Supplementary Figs. A.4 and B.4).

The potential for the publication bias was evaluated through funnel plots (Supplementary Figs. A.2 and B.2) and Egger’s test (Supplementary Figs. A.3 and B.3) for the prevalence of *C. trachomatis* infection among FSWs and MSM. The results indicated that there was no evidence of publication bias in any of the included studies (FSWs: *P* = 0.56; MSM: *P* = 0.84).

## Discussion

Our study presented the most recent and most long-term comprehensive systematic review and meta-analysis of the overall prevalence of *C. trachomatis* infection among FSWs and MSM in China and revealed that the prevalence was very high among these two at-risk populations. A review of 38 eligible studies on FSWs with 29,022 participants revealed a pooled prevalence of *C. trachomatis* infection of 19.5%, and 23 eligible studies on MSM with 8,556 participants showed a pooled prevalence of 12.7% in the rectum, 6.4% in the urethra, and 1.3% in the oropharynx. However, trend analysis did not indicate a statistically significant downwards trend in the prevalence of *C. trachomatis* infection for either population. This research strictly required that the included studies used only NAATs, which have a greater sensitivity than other detection methods and are suitable for various types of clinical specimens. As a result, the findings of this study might be more reliable and accurate than those of previous meta-analyses.

The pooled prevalence of *C. trachomatis* infection in FSWs in our study (19.5%) was lower than that in Papua New Guinea (26.1%) [20] but greater than that in the Middle East and North Africa (14.4%) [21] and Northern Mexico (12.4%) [22]. The estimated prevalence of *C. trachomatis* infection in urethral samples from MSM in our study (6.4%) was significantly greater than that in Germany (2.0%) [23] and was close to the prevalence in Abuja (4.6%) and Lagos (5.4%) in Nigeria [24]. These differences may be related to various influencing factors, such as social, cultural and economic conditions [25].

When we limited our long-term trend analysis to studies utilizing DNA testing, the results indicated a nonsignificant decrease in the prevalence of *C. trachomatis* infection among FSWs from 1998 to 2021. However, an increasing trend in the incidence of *C. trachomatis* infection was reported by the National Notifiable Infectious Disease Surveillance System (NNIDSS) in China from 2008 to 2019 [5, 6]. The NNIDSS compiled data on new cases of genital *C. trachomatis* infection diagnosed by medical institutions, reflecting the incidence rates of the entire population, including both the general public and high-risk groups. The increasing trend in the NNIDSS-reported incidence rates could be attributed to increased awareness among clinicians and the adoption of more sensitive and specific testing methods, leading to an increase in reported cases. Despite the lack of statistical significance, the decrease in the prevalence of *C. trachomatis* infection among FSWs could be partly due to the implementation of the National Syphilis Control and Prevention Plan (2010–2020) and enhanced HIV/AIDS prevention strategies among key populations [26, 27]. These measures might have indirectly influenced the prevalence of *C. trachomatis* infection through comprehensive STD prevention efforts. Our study also did not detect any decreasing trend in the prevalence of *C. trachomatis* infection among MSM from 2003 to 2022. *Neisseria gonorrhoeae* not only causes clinical syndromes similar to *C. trachomatis* infection but also coexists in a significant proportion of patients with *C. trachomatis* infection [28], and another meta-analysis [29] revealed no decreasing trend in the prevalence of gonorrhoea among MSM (2003–2021) in China, which supported our results.

Our study demonstrated that the pooled prevalence of *C. trachomatis* infection among FSWs and MSM significantly varied across different geographical regions within China. The geographic regions with a high prevalence of *C. trachomatis* infection among FSWs were southwestern China (21.7%), central-southern China (20.4%), and eastern China (14.8%). For MSM, the regions with high prevalence rates were northeastern China (7.3%), central-southern China (6.9%), and southwestern China (6.7%). The prevalence of *C. trachomatis* infection among FSWs and MSM differed in different regions. These variations could be attributed to disparities between geographical regions in terms of their socioeconomic status, availability of health services, levels of urbanization, and extent of population migration. In addition, our study revealed that the main provinces surveyed for the prevalence of *C. trachomatis* infection were Yunnan, Jiangsu and Guangdong. Thus, the coverage area of the surveys for high-risk populations of *C. trachomatis* infection in China was quite limited, especially in northern China. To fully estimate the burden of *C. trachomatis* infection among high-risk populations in China, it is necessary to actively carry out relevant surveillance and investigations with reliable detection methods in all regions.

In our study, the prevalence of *C. trachomatis* infection among FSWs based on the specimen collection type was 31.8% for vaginal swabs, 20.6% for cervical swabs, and 14.8% for urine samples. A study aimed at identifying the most effective sample type for *C. trachomatis* infection testing suggested that vaginal swabs were superior to all other sample types and that first-void urine should be avoided [30]. Furthermore, a vaginal swab specimen could be the sample of choice for screening because it not only has high sensitivity and specificity but also requires less processing than urine for most NAATs [31]. The National Center for STD Control and Chinese Center for Disease Control and Prevention issued the National Guidelines for the Prevalence of STDs and Risk Behavior Factors Surveillance Program [32]. The FSW population is regarded as one of the monitored groups, and the use of urine samples during actual surveillance may underestimate the burden of associated STDs. Therefore, we suggest that whenever possible, researchers should opt for vaginal specimens when testing for *C. trachomatis* infection among women. For MSM, our study revealed no difference between urine and urethral samples. This finding aligns with the results of two other studies, which reported similar performance of NAATs on urine and urethral specimens in male populations [33, 34]. The noninvasive collection of specimens has significantly facilitated testing in asymptomatic individuals, making testing more accessible for the MSM population. Hence, urine may be an optimal choice for conducting surveys among MSM.

The rectum was found to have a greater prevalence of *C. trachomatis* infection than the urethra and pharynx among MSM. For instance, a nationwide cross-sectional study conducted in Germany [23] demonstrated that the prevalence of *C. trachomatis* infection was 7.7% in the rectum, 2.0% in the urethra, and 1.1% in the pharynx. A review conducted in the United States [35] revealed that the prevalence of *C. trachomatis* infection was 8.9% in the rectum and 1.7% in the pharynx. Similarly, a study in the United Kingdom [36] revealed that the prevalence of *C. trachomatis* infection in MSM was 6.5% in the anorectum, 4.3% in the urethra, and 1.2% in the pharynx. In our research, the pooled prevalence of *C. trachomatis* infection in the rectum, urethra, and oropharynx was 12.7%, 6.4%, and 1.3%, respectively, among MSM. Rectal and pharyngeal infections play essential roles in the epidemiology of *C. trachomatis* infection, especially in MSM [37], and 57% to 70% of *C. trachomatis* infections are estimated to be missed if only urogenital testing is performed [38–40]. In addition, *C. trachomatis* infections of extragenital sites are often asymptomatic, and individuals with asymptomatic infections may not seek medical care, thereby contributing to the persistence and transmission of STDs [41]. Moreover, rectal *C. trachomatis* infection has been linked to an increased risk of HIV transmission and acquisition [42, 43]. Given the unique predisposition of *C. trachomatis* infection sites in MSM, countries such as the United Kingdom, Australia, and the United States all recommend routine screening for *C. trachomatis* infection in the urethra, pharynx, and anorectum in MSM in their STD prevention and treatment guidelines [44–46]. To effectively reduce the transmission of *C. trachomatis* infection, we strongly suggest testing at multiple anatomical sites in high-risk populations, especially in MSM, in China.

In terms of molecular diagnosis methods, studies that used *C. trachomatis* RNA detection reported a lower prevalence of infection than those that used *C. trachomatis* DNA detection in our study. However, the reasons for this discrepancy remain unclear and deserve further study. We speculated that this may be attributed to the increased susceptibility and degradation of RNA in urine samples. A study conducted on a subset of vaginal swab samples revealed that chlamydial ribosomal RNA was sufficiently stable after long-term storage [47]. Another study demonstrated that storage conditions and the duration of storage barely affected *C. trachomatis* DNA in a negative manner, although frozen urine samples stored for prolonged periods (more than 2 years) could become *C. trachomatis* negative [48]. Consequently, when cross-sectional studies are conducted to assess the prevalence of *C. trachomatis* infection in populations, *C. trachomatis* DNA detection, which produces stable and accurate results, is more suitable. Meanwhile, *C. trachomatis* RNA detection can indicate active infections by pathogens, which makes it more suitable for evaluating disease activity or monitoring the effectiveness of treatment.

## Strengths and limitations

This was the first study that exclusively included studies that used NAATs to systematically review the prevalence of *C. trachomatis* infection in FSWs and MSM in China. We analysed the temporal trends and geographical distributions of *C. trachomatis* infection in these two high-risk populations and also analysed the prevalence of *C. trachomatis* infection depending on the use of different specimen collection types in FSWs, three different specimen collection anatomical sites in MSM and different molecular diagnosis methods among both high-risk populations.

However, this meta-analysis has several limitations. First, there was significant heterogeneity across the included studies. Most participants were recruited via convenience sampling, snowball sampling, or voluntary participation; therefore, the sample was not representative. Second, because of the high cost of NAATs and strict laboratory testing requirements, NAATs are not used in many economically underdeveloped areas. Therefore, most of the studies included in our meta-analysis were performed in economically developed regions, while relevant data from other regions were lacking, and our results may not represent the national prevalence of *C. trachomatis* infection among FSWs and MSM. Third, the FSW population was mostly recruited in entertainment establishments, mainly in urban regions, while the MSM population was primarily recruited from urban bars, parks, and public baths. Meanwhile, data from rural populations were sparse. Given the high migration rates between urban and rural areas, it is important to conduct further research on FSWs and MSM from rural areas to determine their potential role as a bridge facilitating STD transmission from urban to rural regions.

## Conclusion

In conclusion, our study provided the most recent and most long-term estimates of the prevalence of *C. trachomatis* infection among FSWs and MSM in China and showed that the prevalence of *C. trachomatis* infection among these two high-risk populations was high. To reduce the burden of *C. trachomatis* infection in China, it is important to implement effective surveillance and screening strategies for *C. trachomatis* infection among high-risk populations, and particular attention should be given to the selection of specimen types and diagnostic methods, as well as to the detection of extragenital infections.

### Supplementary Information

Supplementary Material 1.

## Data Availability

All data generated or analysed during this study are included in this published article and its supplementary file.

## Abbreviations

*C. trachomatis*: *Chlamydia Trachomatis*
FSWs: Female Sex Workers
MSM: Men Who Have Sex with Men
NAATs: Nucleic Acid Amplification Tests
STDs: Sexually Transmitted Diseases
WHO: World Health Organization
HIV: Human Immunodeficiency Virus
STIs: Sexually Transmitted Infections
AHRQ: Agency for Healthcare Research and Quality
DRI: Detention and Reeducation Institute
CI: Confidence Interval
AIDS: Acquired Immune Deficiency Syndrome
NNIDSS: National Notifiable Infectious Disease Surveillance System
