# Supplementary material for: Prevalence and trends of Chlamydia trachomatis infection in female sex workers and men who have sex with men in China: a systematic review and meta-analysis
Source: BMC Public Health. 2024 Jun 12;24:1579. doi: 10.1186/s12889-024-18804-3 (PMC11170796; doi:10.1186/s12889-024-18804-3)
Supplement: Supplementary file 1 — Supplementary Material 1. [file 12889_2024_18804_MOESM1_ESM.docx]

**Supplementary file published online with the paper.**

**SUPPLEMENTARY MATERIAL**

**Prevalence and trend of *Chlamydia trachomatis* infection in female sex workers and men having sex with men in China: a systematic review and meta-analysis**

**content**

[Supplementary Table A. Preferred Reporting Items for Systematic Reviews and Meta-analyses (PRISMA) checklist (2020) 1](#_Toc142038986)

[Supplementary Table B.1. Data sources and search criteria for systematically reviewing C. trachomatis epidemiology among FSWs and MSM in China 4](#_Toc142038987)

[Supplementary Table B.2. List of studies excluded at full-text screening stage, with brief reasons 5](#_Toc142038988)

[Supplementary Box A. The 12 provinces where the study was published included in our definition of China 11](#_Toc142038989)

[Supplementary Box B. Variables extracted from relevant reports meeting the inclusion criteria 12](#_Toc142038990)

[Supplementary Box C. Definitions of populations classifications 13](#_Toc142038991)

[Supplementary Box D. Factors (variables) selected a priori and included in univariable and multivariable meta-regression analyses 14](#_Toc142038992)

[Supplementary Table C.1. Studies reporting prevalence of C. trachomatis among FSWs in China 15](#_Toc142038993)

[Supplementary Table C.2. Studies reporting prevalence of C. trachomatis among MSM in China 19](#_Toc142038994)

[Supplementary Table D. AHRQ cross-sectional quality evaluatio 23](#_Toc142038995)

[Supplementary Figure A. and B. Forest plots presenting outcomes of the pooled prevalence among FSWs (Fig. A) and MSM (Fig. B) in China 28](#_Toc142038996)

[Fig.A.1. The pooled prevalence of C. trachomatis in FSWs 28](#_Toc142038997)

[Fig.A.2. Funnel Chart in FSWs 29](#_Toc142038998)

[Fig.A.3. Egger’s test in FSWs 29](#_Toc142038999)

[Fig.A.4. Sensitivity Analysis in FSWs 30](#_Toc142039000)

[Fig.B.1. The pooled prevalence of C. trachomatis in MSM 31](#_Toc142039001)

[Fig.B.2. Funnel Chart in MSM 31](#_Toc142039002)

[Fig.B.3. Egger’s Test in MSM 32](#_Toc142039003)

[Fig.B.4. Sensitivity Analysis in MSM 32](#_Toc142039004)

[Supplementary Figure C. and D. Forest plots presenting outcomes of the pooled prevalence among FSWs and MSM in China by Subgroup analysis 33](#_Toc142039005)

[Fig.C.1. The pooled prevalence among FSWs in the subgroup analysis of sample size 33](#_Toc142039006)

[Fig.C.2. The pooled prevalence among FSWs in the subgroup analysis of publication language 34](#_Toc142039007)

[Fig.C.3. The pooled prevalence among FSWs in the subgroup analysis of data collection period 35](#_Toc142039008)

[Fig.C.4. The pooled prevalence among FSWs in the subgroup analysis of study region 36](#_Toc142039009)

[Fig.C.5. The pooled prevalence among FSWs in the subgroup analysis of study specimen collection types 37](#_Toc142039010)

[Fig.C.6. The pooled prevalence among FSWs in the subgroup analysis of study molecular diagnosis methods 38](#_Toc142039010)

[Fig.C.7. The pooled prevalence among FSWs in the subgroup analysis of study recruitment sites 39](#_Toc142039011)

[Fig.D.1. The pooled prevalence among MSM in the subgroup analysis of sample size 40](#_Toc142039012)

[Fig.D.2. The pooled prevalence among MSM in the subgroup analysis of publication language 41](#_Toc142039013)

[Fig.D.3. The pooled prevalence among MSM in the subgroup analysis of data collection period 42](#_Toc142039014)

[Fig.D.4. The pooled prevalence among MSM in the subgroup analysis of study region 43](#_Toc142039015)

[Fig.D.5. The pooled prevalence among MSM in the subgroup analysis of specimen collection types 44](#_Toc142039016)

[Fig.D.6. The pooled prevalence among MSM in the subgroup analysis of molecular diagnosis methods 45](#_Toc142039016)

[Fig.D.7. The pooled prevalence among MSM in the subgroup analysis of specimen collection anatomical sites 46](#_Toc142039017)

[Supplementary Table E. Sensitivity analyses with restricted studies in specific conditions 47](#_Toc142039018)

[References 48](#_Toc142039019)

# Supplementary Table A. Preferred Reporting Items for Systematic Reviews and Meta-analyses (PRISMA) checklist (2020)

| **Section and Topic** | **Item #** | **Checklist item** | **Location where item is reported** |
| --- | --- | --- | --- |
| **TITLE** | | |  |
| Title | 1 | Identify the report as a systematic review. | p. 1 |
| **ABSTRACT** | | |  |
| Abstract | 2 | See the PRISMA 2020 for Abstracts checklist. | p. 1 |
| **INTRODUCTION** | | |  |
| Rationale | 3 | Describe the rationale for the review in the context of existing knowledge. | p. 1 |
| Objectives | 4 | Provide an explicit statement of the objective(s) or question(s) the review addresses. | p. 1 |
| **METHODS** | | |  |
| Eligibility criteria | 5 | Specify the inclusion and exclusion criteria for the review and how studies were grouped for the syntheses. | p. 3 |
| Information sources | 6 | Specify all databases, registers, websites, organisations, reference lists and other sources searched or consulted to identify studies. Specify the date when each source was last searched or consulted. | p. 3 |
| Search strategy | 7 | Present the full search strategies for all databases, registers and websites, including any filters and limits used. | Supplement Table B.1 |
| Selection process | 8 | Specify the methods used to decide whether a study met the inclusion criteria of the review, including how many reviewers screened each record and each report retrieved, whether they worked independently, and if applicable, details of automation tools used in the process. | p. 3 |
| Data collection process | 9 | Specify the methods used to collect data from reports, including how many reviewers collected data from each report, whether they worked independently, any processes for obtaining or confirming data from study investigators, and if applicable, details of automation tools used in the process. | p. 3 |
| Data items | 10a | List and define all outcomes for which data were sought. Specify whether all results that were compatible with each outcome domain in each study were sought (e.g. for all measures, time points, analyses), and if not, the methods used to decide which results to collect. | Supplement Box B |
|  | 10b | List and define all other variables for which data were sought (e.g. participant and intervention characteristics, funding sources). Describe any assumptions made about any missing or unclear information. | NA |
| Study risk of bias assessment | 11 | Specify the methods used to assess risk of bias in the included studies, including details of the tool(s) used, how many reviewers assessed each study and whether they worked independently, and if applicable, details of automation tools used in the process. | p. 3 |
| Effect measures | 12 | Specify for each outcome the effect measure(s) (e.g. risk ratio, mean difference) used in the synthesis or presentation of results. | p. 4 |
| Synthesis methods | 13a | Describe the processes used to decide which studies were eligible for each synthesis (e.g. tabulating the study intervention characteristics and comparing against the planned groups for each synthesis (item #5)). | p. 3-4 |
|  | 13b | Describe any methods required to prepare the data for presentation or synthesis, such as handling of missing summary statistics, or data conversions. | p. 4 |
|  | 13c | Describe any methods used to tabulate or visually display results of individual studies and syntheses. | NA |
|  | 13d | Describe any methods used to synthesize results and provide a rationale for the choice(s). If meta-analysis was performed, describe the model(s), method(s) to identify the presence and extent of statistical heterogeneity, and software package(s) used. | p. 4 |
|  | 13e | Describe any methods used to explore possible causes of heterogeneity among study results (e.g. subgroup analysis, meta-regression). | p. 4 |
|  | 13f | Describe any sensitivity analyses conducted to assess robustness of the synthesized results. | p. 4 |
| Reporting bias assessment | 14 | Describe any methods used to assess risk of bias due to missing results in a synthesis (arising from reporting biases). | p. 4 |
| Certainty assessment | 15 | Describe any methods used to assess certainty (or confidence) in the body of evidence for an outcome. | p. 3 |
| **RESULTS** | | |  |
| Study selection | 16a | Describe the results of the search and selection process, from the number of records identified in the search to the number of studies included in the review, ideally using a flow diagram. | p. 5; Figure 1 |
|  | 16b | Cite studies that might appear to meet the inclusion criteria, but which were excluded, and explain why they were excluded. | Supplementary  Table B.2 |
| Study characteristics | 17 | Cite each included study and present its characteristics. | Supplementary  Tables C.1 and C.2 |
| Risk of bias in studies | 18 | Present assessments of risk of bias for each included study. | NA |
| Results of individual studies | 19 | For all outcomes, present, for each study: (a) summary statistics for each group (where appropriate) and (b) an effect estimate and its precision (e.g. confidence/credible interval), ideally using structured tables or plots. | Supplementary  Firgures A. and B. |
| Results of syntheses | 20a | For each synthesis, briefly summarise the characteristics and risk of bias among contributing studies. | Supplementary  Tables C.1 and C.2 |
|  | 20b | Present results of all statistical syntheses conducted. If meta-analysis was done, present for each the summary estimate and its precision (e.g. confidence/credible interval) and measures of statistical heterogeneity. If comparing groups, describe the direction of the effect. | Supplementary  Firgures A. B. C. and D. |
|  | 20c | Present results of all investigations of possible causes of heterogeneity among study results. | P 7-10 Table 1. 2. |
|  | 20d | Present results of all sensitivity analyses conducted to assess the robustness of the synthesized results. | Supplementary Table E. |
| Reporting biases | 21 | Present assessments of risk of bias due to missing results (arising from reporting biases) for each synthesis assessed. | NA |
| Certainty of evidence | 22 | Present assessments of certainty (or confidence) in the body of evidence for each outcome assessed. | Supplementary  Firgures A. B. C. and D. |
| **DISCUSSION** | | |  |
| Discussion | 23a | Provide a general interpretation of the results in the context of other evidence. | p. 13 |
|  | 23b | Discuss any limitations of the evidence included in the review. | p. 16 |
|  | 23c | Discuss any limitations of the review processes used. | NA |
|  | 23d | Discuss implications of the results for practice, policy, and future research. | p. 14-16 |
| **OTHER INFORMATION** | | |  |
| Registration and protocol | 24a | Provide registration information for the review, including register name and registration number, or state that the review was not registered. | p. 2 |
|  | 24b | Indicate where the review protocol can be accessed, or state that a protocol was not prepared. | p. 2 |
|  | 24c | Describe and explain any amendments to information provided at registration or in the protocol. | NA |
| Support | 25 | Describe sources of financial or non-financial support for the review, and the role of the funders or sponsors in the review. | p. 17 |
| Competing interests | 26 | Declare any competing interests of review authors. | p. 17 |
| Availability of data, code and other materials | 27 | Report which of the following are publicly available and where they can be found: template data collection forms; data extracted from included studies; data used for all analyses; analytic code; any other materials used in the review. | p. 17 |

Abbreviations: NA = Not applicable, p = page.

*From:* Page MJ, McKenzie JE, Bossuyt PM, et al. The PRISMA 2020 statement: an updated guideline for reporting systematic reviews. BMJ 2021;372:n71.

https://doi.org/10.1136/bmj.n71

For more information, visit: <http://www.prisma-statement.org/>

# Supplementary Table B.1. Data sources and search criteria for systematically reviewing *C. trachomatis* epidemiology among FSWs and MSM in China

| **PubMed (last searched April 30, 2023):101** |
| --- |
| ("Chlamydia trachomatis"[Mesh] OR " Chlamydia"[Mesh] OR Chlamydia [Title/Abstract] OR Chlamydia trachomatis [Title/Abstract]) AND ("China"[Mesh] OR China [Title/Abstract] OR Chinese[Title/Abstract]) AND ("Prevalence"[Mesh] OR "Epidemiology"[Mesh] OR "Infections"[Mesh] OR "Cross-Sectional Studies"[Mesh] OR Prevalence[Title/Abstract] OR Epidemiology[Title/Abstract] OR Infections[Title/Abstract] OR Cross-Sectional Studies[Title/Abstract] OR survey[Title/Abstract]) AND ("Sex Workers"[Mesh] OR "Homosexuality, Male"[Mesh] OR Men who have sex with men[Title/Abstract] OR MSM[Title/Abstract] OR Female sex workers[Title/Abstract] OR FSWs[Title/Abstract]) |
|  |
|  |
|  |
| **Web of Science (last searched April 30, 2023):144** |
| (TS=(Chlamydia trachomatis)) OR TS=(Chlamydia) and (((TS=(Prevalence)) OR TS=(Epidemiology)) OR TS=(Infections)) OR TS=(Cross-Sectional Studies) and (((((TS=(female sex worker)) OR TS=(FSWs)) OR TS=(Sex Workers)) OR TS=(Homosexuality)) OR TS=(Men who have sex with men)) OR TS=(MSM) and TS=(China) |
| **China National Knowledge Infrastructure (CNKI) (last searched April 30, 2023): 1359** |
| (TS= (Chlamydia trachomatis OR CT) AND TS= (China OR Chinese) AND TS= (Prevalence OR Epidemiology OR Infections OR Cross-Sectional Study OR Survey) AND TS= (Female sex workers OR Men who have sex with men OR FSWs OR MSM) |
|  |
| **Wan-fang Data (last searched April 30, 2023): 182** |
| (TS= (Chlamydia trachomatis OR CT) AND TS= (China OR Chinese) AND TS= (Prevalence OR Epidemiology OR Infections OR Cross-Sectional Study OR Survey) AND TS= (Female sex workers OR Men who have sex with men OR FSWs OR MSM) |
|  |
| **Chinese Scientific Journals Database (VIP) (last searched April 30, 2023): 103** |
| (TS= (Chlamydia trachomatis OR CT) AND TS= (China OR Chinese) AND TS= (Prevalence OR Epidemiology OR Infections OR Cross-Sectional Study) AND TS= (Female sex workers OR Men who have sex with men OR FSWs OR MSM) |
|  |
|  |

Abbreviations: TS: Title/Abstract, FSWs = Female sex workers, MSM = Men who have sex with men

# Supplementary Table B.2. List of studies excluded at full-text screening stage, with brief reasons

| **NO.** | **Author, year** | **Title** | **Language of publication** | **Database sources** | **Reasons** |
| --- | --- | --- | --- | --- | --- |
| 1 | Chen et al,2010 | Investigation on HIV and STI Infections among Men Who Have Sex with Men in Nanning City during 2006 - 2008 | Chinese | CNKI | NAATs were not used |
| 2 | Wang et al,2008 | Prevalence of HIV/STD and risk behavior among men who have sex with men in Shenyang | Chinese | CNKI | NAATs were not used |
| 3 | Zhang et al,2007 | Risk factors of HIV infection and prevalence of co-infections among men who have sex with men in Beijing, China | English | PubMed | NAATs were not used |
| 4 | Wang et al,2020 | Screening for chlamydia trachomatis and Neisseria gonorrhoeae infections in men who have sex with men | Chinese | VIP | Participants did not meet the inclusion criteria |
| 5 | Wang et al,2011 | The epidemics of AIDS and STDs among MSM | Chinese | CNKI | Did not have the required data |
| 6 | Li,2006 | Study on the Relationship Between HIV/AIDS High Risk Behavior and STD among Men Who Have Sex with Men in Mainland Chine | Chinese | CNKI | Did not have the required data |
| 7 | Li et al,2009 | A study on the relationship between sexual partners of 2250 male contacts and sexually transmitted infection rate | Chinese | CNKI | Did not have the required data |
| 8 | Ma et al,2010 | Common sexually transmitted diseases among men who have contact with men | Chinese | CNKI | Did not have the required data |
| 9 | Shi et al,2003 | STI status and associated high-risk sexual behaviors among male-to-male contacts | Chinese | Wan Fang | Did not have the required data |
| 10 | Wang et al,2008 | Knowledge of HIV/AIDS STD prevention and treatment and STD infection among male contacts in Guangling District of Yangzhou City | Chinese | VIP | Did not have the required data |
| 11 | Sun et al,2012 | HIV-related behavior and STD infection status among MSM in Beijing | Chinese | CNKI | Did not have the required data |
| 12 | Yu et al,2013 | Prevalence of AIDS-related high-risk behaviors among men who have sex with men with STI | Chinese | Wan fang | Did not have the required data |
| 13 | Wang et al,2013 | Behavioral characteristics and HIV/STD infection among men who have sex with men in Yichang City | Chinese | CNKI | Did not have the required data |
| 14 | Zhang et al,2008 | A survey of HIV/AIDS related behaviors among 2250 MSM in nine major cities of China | Chinese | Wan fang | Did not have the required data |
| 15 | Zheng et al,2006 | Research progress on HIV/STIs infection status and high-risk sex behavior among Chinese male male-to-male contacts | Chinese | Wan fang | Did not have the required data |
| 16 | Jiang et al,2022 | Prevalence of anatomically specific infections with Chlamydia trachomatis among men who have sex with men in China: protocol for a nationwide cross-sectional study as part of Disease Burden Surveillance of Infections with Chlamydia (DBSIC) | English | PubMed | Did not have the required data |
| 17 | Liu et al,2014 | Analysis of multisite infection of Mycoplasma genitalium and chlamydia trachomatis in male-to-male contacts | Chinese | Wan fang | Did not describe CT testing method |
| 18 | Tan et al,2009 | Study on Sexual Behavior ，HIV and Sexual Transmitted Diseases Among Men Who Have Sex with Men (MSM) in Shenzhen | Chinese | CNKI | Did not describe CT testing method |
| 19 | Tao et al,2018 | Investigation on risk factors of STD/AIDS among MSM in Cheng-guan district of Lanzhou | Chinese | Wan fang | Did not describe CT testing method |
| 20 | Xu et al,2012 | Investigation on Other Infectious Diseases in Men Who Have Sex with Men, with Acquired Immune Deficiency Syndrome in Chengdu City | Chinese | Wan fang | Did not describe CT testing method |
| 21 | Zhou,2019 | Molecular epidemiology of Chlamydia trachomatis infections among men who have sex with men and meta-analysis of point-of-care tests for chlamydia | English | PubMed | Did not have the required data |
| 22 | Li et al,2011 | Prevalence of anorectal Chlamydia trachomatis infection and its genotype distribution among men who have sex with men in Shenzhen, China | English | PubMed | Full text unavailable |
| 23 | Li et al,2018 | Analysis on status and risk factors of sexually transmitted diseases among the HIV-positive MSM, Jinan city, 2016 | Chinese | Wan fang | Participants did not meet the inclusion criteria |
| 24 | Liu,2017 | Prevalence and risk factors of sexually transmitted Diseases among MSM infected with HIV | Chinese | CNKI | Participants did not meet the inclusion criteria |
| 25 | Lin et al,2021 | High prevalence of sexually transmitted coinfections among at-risk people living with HIV | English | PubMed | Participants did not meet the inclusion criteria |
| 26 | Dai et al,2009 | Investigation and Analysis on Sexually Transmitted Infection Among Different Females Group in Guangzhou | Chinese | CNKI | NAATs were not used |
| 27 | Jiang et al,2012 | Survey of STI/AIDS-related knowledge, attitude and behaviors and infection rates of sex workers in entertainment places in Jingzhou City | Chinese | CNKI | NAATs were not used |
| 28 | Jiang et al,2009 | Seroepidemiological Study on Infection with Chlamydia trachomatis, Mycoplasma hominis and Mycoplasma genitalium in Commercial Sex Workers in Urumqi | Chinese | CNKI | NAATs were not used |
| 29 | Li et al,2006 | Study of HIV-risk factors among commercial sex workers in four cities of Xinjiang | Chinese | CNKI | NAATs were not used |
| 30 | Li et al,2009 | Prevalence of HIV infection and sexually transmitted diseases and associated risk factors among female sex workers in Guangdong province | Chinese | VIP | NAATs were not used |
| 31 | Liang et al,2003 | Survey of infectious status of sexually transmitted diseases in 421 girls working in public and entertainment places in Danzhou City | Chinese | CNKI | NAATs were not used |
| 32 | Ma et al,2016 | Investigation on sexually transmitted diseases among prostitutes in Yizheng City | Chinese | CNKI | NAATs were not used |
| 33 | Mei et al,2009 | Investigation on STD prevalence and intervention effect of sex workers in 4 cities of Shanxi Province | Chinese | CNKI | NAATs were not used |
| 34 | Ni et al,2011 | Surveillance on STD infection of some sex workers and their clients in Haiyan County during 2004 - 2008 | Chinese | Wan fang | NAATs were not used |
| 35 | Shu et al,2011 | Detection of chlamydia trachomatis in reproductive tract of 225 female prostitutes | Chinese | Wan fang | NAATs were not used |
| 36 | Wu et al,2002 | Surveillance and Intervention Study of Sexual Transmitted Diseases in Commercial Female Sex Workers | Chinese | CNKI | NAATs were not used |
| 37 | Yan et al,2004 | A survey on high-risk behavior and main sexually transmitted diseases among prostitutes in Jiangsu | Chinese | CNKI | NAATs were not used |
| 38 | Yang et al,2011 | Status of STDs Epidemic Among Female Sexual Workers in Chong ‘a District in Wuxi | Chinese | CNKI | NAATs were not used |
| 39 | Yang et al,2009 | Study on prevalence of sexually transmitted infections among female sex workers in part areas of Yunnan | Chinese | CNKI | NAATs were not used |
| 40 | Ye et al,2008 | Investigation on Chlamydia trachomatis prevalence among different female population groups in Guangzhou and related risk factors | Chinese | CNKI | NAATs were not used |
| 41 | Yu,2014 | Investigation and analysis of the sexually transmitted disease situation among 812 female sex workers | Chinese | CNKI | NAATs were not used |
| 42 | Zhang et al,2012 | Survey and Analysis on STDs of 1 286 Female Sex Workers in Jiangmen City of Guangdong Province | Chinese | Wan fang | NAATs tests were not used |
| 43 | Zhang et al,2014 | Prevalence of sexual transmitted diseases among 663 female sex workers | Chinese | CNKI | NAATs were not used |
| 44 | Zhang et al,2012 | An epidemiological study on HIV/STD infection in female sex workers in Baotou city | Chinese | CNKI | NAATs were not used |
| 45 | Zheng et al,2011 | The Current Infection Situation of Human Papilloma Virus (HPV) and Other Sexually Transmitted Disease (STD) among the Gynecology Clinic Clients and the Sex Workers in Reeducation Center in Tianjin | Chinese | CNKI | NAATs were not used |
| 46 | Zhu et al,2012 | Analysis of STI/HIV infection among sex workers in entertainment venues in Guangxi | Chinese | CNKI | NAATs were not used |
| 47 | Hong et al,2011 | Factors associated with sexually transmitted infection underreporting among female sex workers in China | English | PubMed | NAATs were not used |
| 48 | A van den Hoek et al,2001 | High prevalence of syphilis and other sexually transmitted diseases among sex workers in China: potential for fast spread of HIV | English | PubMed | NAATs were not used |
| 49 | Zhu et al,2012 | Epidemiology of sexually transmitted infections, HIV, and related high-risk behaviors among female sex workers in Guangxi Autonomous Region, China | English | PubMed | NAATs were not used |
| 50 | Cao et al,2006 | Sexually transmitted diseases and risk factors among female sex workers in a heavy drug using area | Chinese | CNKI | Did not have the required data |
| 51 | Chen et al,2009 | Survey of HIV/STD risk behavior and infection among street sex workers | Chinese | CNKI | Did not have the required data |
| 52 | Ding,2013 | HIV Infection and Risk F actors in Female Sex Workers in Kaiyuan City, Yunnan Province | Chinese | CNKI | Did not have the required data |
| 53 | He et al,2014 | Analysis of Sexually Transmitted Infections among Female Sex Workers and Their Clients in Guangzhou City | Chinese | CNKI | Did not have the required data |
| 54 | Huang et al,2015 | STD/HIV Infection and Behavioral Characteristics Among High-Risk Group Sentinel Surveillance, Shandong Province,2013 | Chinese | CNKI | Did not have the required data |
| 55 | Jia et al,2021 | Analysis on the physical examination results of sex workers in entertainment venues in Jianye District, Nanjing | Chinese | Wan fang | Did not have the required data |
| 56 | Li et al,2012 | Survey Of high-risk behaviors and HIV/STD infection among low-grade FSWs In Guigang City | Chinese | CNKI | Did not have the required data |
| 57 | Li et al,2013 | Survey on HIV/STIs Prevalence and High-Risk Behaviors Among Female Sex Workers in Different Settings | Chinese | CNKI | Did not have the required data |
| 58 | Qin et al,2012 | Analysis on effects of the Aids/reproductive tract infections intervention to female sex workers in low-class establishments of Maanshan | Chinese | VIP | Did not have the required data |
| 59 | Xia et al,2009 | Prevalence of self- reported symptoms of reproductive tract infection among female sex workers in Beijing | Chinese | VIP | Did not have the required data |
| 60 | Zhang et al,2006 | Analysis on the effect of prevention and treatment of sexually transmitted AIDS among prostitutes at low level places | Chinese | VIP | Did not have the required data |
| 61 | Yang et al,2004 | Study on the treatment of sexually transmitted diseases among sex workers in a coastal city | Chinese | Wan fang | Did not have the required data |
| 62 | Yuan et al,2008 | Investigation on Sexually Transmitted Diseases and Medical Behaviors among Female Commercial Sexy Workers on the Border between Urban and Rural | Chinese | CNKI | Did not have the required data |
| 63 | Nong et al,2008 | Investigation on 406 prostitutes in Ningming County in 2007 | Chinese | VIP | Did not have the required data |
| 64 | Chen et al,2005 | Sexually transmitted infections among female sex workers in Yunnan, China | English | PubMed | Did not have the required data |
| 65 | Liu et al,2013 | Investigation on knowledge of AIDS prevention and treatment and STD infection among 193 prostitutes | Chinese | CNKI | Did not describe CT testing method |
| 66 | Luo et al,2015 | Prevalence of HIV and sexually transmitted infections among female sex workers in different work sites of a city in Fujian Province | Chinese | CNKI | Did not describe CT testing method |
| 67 | Tao et al,2013 | Prevalence survey of STD/AIDS in unlicensed prostitute in Chengguan District of Lanzhou City | Chinese | CNKI | Did not describe CT testing method |
| 68 | Gao et al,2007 | Molecular Epidemiological Studies on Genital Infections with Chlamydia trachomatis in Women at High Risk | Chinese | CNKI | Did not have the required data |
| 69 | Zhao et al,2011 | Research on Aids/Infectious Diseases of Genital Tract and Related Behavior of Female Sex Workers in Low-grade Areas | Chinese | VIP | Did not have the required data |
| 70 | Zhong et al,2011 | HIV/STD infection and risk behaviors among commercial sex workers at various places in Wuzhou City, Guangxi Zhuang Autonomous Region | Chinese | CNKI | Did not have the required data |
| 71 | Gao et al,2007 | Distribution study of Chlamydia trachomatis serovars among high-risk women in China performed using PCR-restriction fragment length polymorphism genotyping | English | PubMed | Did not have the required data |
| 72 | Shi et al,2007 | Epidemiological survey of female sex service workers in Baotou area | Chinese | CNKI | Scientific and technological achievements report |
| 73 | Liu,2019 | Investigation of the Chlamydia trachomatis and Neisseria gonorrhoeae infection and influencing factors among female sex workers in an area of Yunnan Province | Chinese | CNKI | Did not describe the survey year |
| 74 | Chen et al,2006 | Prevalence of Chlamydia trachomatis infections among women from different settings in China: implications for STD surveillance | English | PubMed | Did not describe the survey year |
| 75 | Yao,2010 | Serial cross-sectional studies of HIV/STIs infections among Female Sex Workers in one City of Yunnan Province | Chinese | CNKI | Same study sample |
| 76 | Yin et al,2013 | Association of sexually transmitted infections with high-risk human papillomavirus types: a survey with 802 female sex workers in China | English | PubMed | Same study sample |
| 77 | Ni et al,1996 | A seroepidemiologic study of Chlamydia pneumoniae, Chlamydia trachomatis and Chlamydia psittaci in different populations on the mainland of China. | English | Web of Science | NAATs were not used |
| 78 | Qin et al,2016 | Prevalence of Chlamydia trachomatis Genotypes in Men Who Have Sex with Men and Men Who Have Sex with Women Using Multilocus VNTR Analysis-ompA Typing in Guangzhou, China | English | Web of Science | Did not have the required data |
| 79 | Zhou et al, 2012 | Survey of prevalence and behavioral risk factors for AIDS/STD among female sex workers in Tanjn | Chinese | CNKI | NAATswere not used |

# Supplementary Box A. The 12 provinces where the study was published included in our definition of China

• **Northwestern China:** Gansu

• **Southwestern China:** Yunnan

• **Eastern China:** Jiangsu, Jiangxi, Zhejiang, Shanghai

• **Northeastern China:** Jilin, Liaoning

• **Central-southern China**: Hubei, Guangdong, Guangxi, Hainan

# Supplementary Box B. Variables extracted from relevant reports meeting the inclusion criteria

1. Author(s)

2. Publication title

3. Publication year

4. Publication language

5. Year(s) of data collection

6. Provincial districts of survey

7. Study population

8. Study design

9. Sample size

10. Number of positive C. trachomatis infection

11. Laboratory testing methods

12. Specimen collection types

13. Molecular diagnosis methods

14. Recruitment sites (FSWs)

15. Specimen collection anatomical sites (MSM)

Abbreviations: C. trachomatis = Chlamydia trachomatis

# Supplementary Box C. Definitions of populations classifications

1. **Higher-risk populations**: These include populations at high risk of exposure

to C. trachomatis because of specific sexual risk behaviors such as

female sex workers (FSWs), men who have sex with men (MSM).

2. **FSWs**: In general, we define FSWs as individuals who are biologically female,

aged 16 years or older, and have self-reported providing vaginal, oral, or anal sex

services for male clients within the past 12 months.

3. **MSM:** In general, we define MSM as individuals who were assigned male at birth,

are 18 years of age or older, and have self-reported engaging in anal or oral sex with

another male in the past year.

Abbreviations: C. trachomatis = Chlamydia trachomatis

# Supplementary Box D. Factors (variables) selected a priori and included in univariable and multivariable meta-regression analyses

1. Sample size:

o <572 (FSWs)

o ≥572 (FSWs)

o <293 (MSM)

o ≥293 (MSM)

1. Publication language:

o English

o Chinese

1. Data collection period:

o 1998-2004 (FSWs)

o 2005-2009 (FSWs)

o 2010-2015 (FSWs)

o 2016-2021 (FSWs)

o 2003-2009 (MSM)

o 2010-2015 (MSM)

o 2016-2022 (MSM)

1. Study region

o Eastern China

o Northern China

o Northwestern China

o Northeastern China

o Southwestern China

o Central-south China

1. Specimen collection types:

o Cervical swab (FSWs)

o Vaginal swab (FSWs)

o Urine (FSWs)

o Urethral swab (MSM)

o Urine (MSM)

1. Molecular diagnosis methods:

o DNA

o RNA

1. Recruitment sites (FSWs):

o Entertainment venues

o Detention and reeducation institutes

1. Specimen collection anatomical sites (MSM):

o Urethra

o Rectum

o Oropharynx

1. Study quality as defined in Supplementary Table D:

o Moderate

o High

# Supplementary Table C.1. Studies reporting prevalence of *C. trachomatis* among FSWs in China

| **Author,**  **year** | **Study type** | **Dates** | **Region** | **Laboratory testing methods**  **(molecular)** | **Recruitment site** | **Specimen**  **collection types** | **Participants Size** | **Number of positive persons** | **Prevalence of C. trachomatis** | **Study Quality** | **Language of publication** |
| --- | --- | --- | --- | --- | --- | --- | --- | --- | --- | --- | --- |
| C. trachomatis infection of the genital tract | | | | | | | | | | | |
| Peng et al  (2004) 1 | Cross sectional | 2003 | Guangdong | PCR (DNA) | reception and reeducation institutes | Cervical swab | 110 | 26 | 23.64% | High | Chinese |
| Yang et al  (2004) 2 | Cross sectional | 2002 | Guangdong | PCR (DNA) | reception and reeducation institutes | Cervical swab | 130 | 42 | 32.31% | High | Chinese |
| Yang et al  (2006) 3 | Cross sectional | 2004 | Hubei | PCR (DNA) | Entertainment venues | Cervical swab | 577 | 82 | 14.21% | Moderate | Chinese |
| Zhu et al  (2006) 4 | Cross sectional | 2003-2004 | Guangdong | PCR (DNA) | reception and reeducation institutes | Cervical swab | 380 | 110 | 28.95% | Moderate | Chinese |
| Wang et al  (2007) 5 | Cross sectional | 2006 | Yunnan | PCR (DNA) | Entertainment venues | Cervical swab | 832 | 235 | 28.25% | High | Chinese |
| Gao  (2007) 6 | Cross sectional | 2005-2006 | Yunnan | PCR (DNA) | Entertainment venues | Cervical swab | 229 | 35 | 15.28% | Moderate | Chinese |
| Zhang  (2007) 7 | Cross sectional | 2006 | Yunnan | PCR (DNA) | Entertainment venues | Urine | 160 | 75 | 46.88% | Moderate | Chinese |
| Xu et al  (2008) 8 | Cross sectional | 2006 | Yunnan | PCR (DNA) | Entertainment venues | Cervical swab | 95 | 44 | 46.32% | High | English |
| Wang et al  (2008) 9 | Cross sectional | 2006 | Yunnan | PCR (DNA) | Entertainment venues | Cervical swab | 737 | 191 | 25.92% | High | Chinese |
| Jin  (2010) 10 | Cross sectional | 2007 | Yunnan | PCR (DNA) | Entertainment venues | Cervical swab | 696 | 126 | 18.10% | High | Chinese |
| Zhong et al  (2010) 11 | Cross sectional | 2009 | Guangxi | PCR (DNA) | Entertainment venues | Cervical swab | 406 | 88 | 21.67% | High | Chinese |
| Jin et al  (2011) 12 | Cross sectional | 2008 | Yunnan | PCR (DNA) | Entertainment venues | Cervical swab | 568 | 99 | 17.43% | High | English |
| Li et al  (2011) 13 | Cross sectional | 2009-2010 | Zhejiang | PCR (DNA) | other | Cervical swab | 98 | 40 | 40.82% | Moderate | Chinese |
| Feng  (2012) 14 | Cross sectional | 2009 | Yunnan | PCR (DNA) | Entertainment venues | Cervical swab | 113 | 12 | 10.62% | High | Chinese |
| Zhang et al  (2012) 15 | Cross sectional | 2009 | Jiangsu | PCR (DNA) | Entertainment venues | Cervical swab | 848 | 125 | 14.74% | High | Chinese |
| Han et al  (2013) 16 | Cross sectional | 2009-2010 | Guangxi | PCR (DNA) | Entertainment venues | Cervical swab | 805 | 161 | 20.0% | High | Chinese |
| Chen et al  (2013) 17 | Cross sectional | 2009 | Jiangsu  Guangxi  Guangdong  Hainan | PCR (DNA) | Entertainment venues | Cervical swab | 3099 | 536 | 17.30% | High | English |
| Han et al  (2014) 18 | Cross sectional | 2009 | Guangdong  Hainan | PCR (DNA) | Entertainment venues | Cervical swab | 989 | 200 | 20.22% | High | English |
| Tang et al  (2014) 19 | Cross sectional | 2009 | Jiangsu | PCR (DNA) | Entertainment venues | Cervical swab | 849 | 124 | 14.61% | High | English |
| Xu et al  (2014) 20 | Cross sectional | 2009-2010 | Hainan | PCR (DNA) | Entertainment venues | N/A | 402 | 71 | 17.66% | Moderate | Chinese |
| Li et al  (2015) 21 | Cross sectional | 2010-2011 | Hubei  Guangdong  Gansu | PCR (DNA) | other | Cervical swab | 1033 | 193 | 18.68% | High | English |
| Luo et al  (2015) 22 | Cross sectional | 2012 | Yunnan | PCR (DNA) | Entertainment venues | Cervical swab | 833 | 235 | 28.21% | High | English |
| Fan et al  (2016) 23 | Cross sectional | 2009-2010 | Hainan | PCR (DNA) | Entertainment venues | Cervical swab | 413 | 65 | 15.74% | Moderate | Chinese |
| Guo et al  (2017) 24 | Cross sectional | 2010-2011 | Jiangsu | PCR (DNA) | Entertainment venues | Cervical swab | 436 | 74 | 16.97% | High | English |
| Li et al  (2017) 25 | Cross sectional | 2008-2009 | Jining  Gansu  Hubei  Zhejiang  Guangdong | PCR (DNA) | Entertainment venues | Cervical swab | 1607 | 222 | 13.81% | Moderate | English |
| Yang et al  (2020) 26 | Cross sectional | 2017-2018 | Yunnan | PCR (DNA) | Entertainment venues | Vaginal swab | 423 | 56 | 13.24% | High | Chinese |
| Ma et al  (2022) 27 | Cross sectional | 2020 | Yunnan | PCR (DNA) | Entertainment venues | Urine | 200 | 34 | 17.00% | High | Chinese |
| Chen et al  (2022) 28 | Cross sectional | 2018-2020 | Zhejiang | PCR (RNA) | Entertainment venues | Urine | 375 | 24 | 6.40% | Moderate | Chinese |
| Dong et al  (2021) 29 | Cross sectional | 2019 | Yunnan | PCR (DNA) | Entertainment venues | Urine/ Cervical swab | 1005 | 145 | 14.43% | High | Chinese |
| Qi et al  (2022)30 | Cross sectional | 2020 | Guangdong | PCR (DNA) | Entertainment venues | Urine | 250 | 55 | 22.00% | Moderate | Chinese |
| Zha et al (2011)31 | Cross sectional | 2009 | Shanghai | LCR (DNA) | Entertainment venues | Urine | 125 | 26 | 20.80% | Moderate | Chinese |
| Li et al  (2009)32 | Cross sectional | 2006 | Yunnan | PCR (DNA) | Entertainment venues | Cervical swab | 734 | 147 | 20.03% | High | Chinese |
| Chen et al  (2005)33 | Cross sectional | 1999-2000 | Yunnan | PCR (DNA) | Reeducation institutes | Vaginal swab | 505 | 296 | 58.60% | Moderate | English |
| Shi et al  (2022)34 | Cross sectional | 2021 | Jiangsu | PCR (RNA) | Entertainment venues | Urine | 3307 | 141 | 4.26% | High | English |
| Wang et al  (2009)35 | Cross sectional | 2006 | Yunnan | PCR (DNA) | Entertainment venues | Cervical swabs | 737 | 191 | 25.92% | High | English |
| Remis et al  (2014)36 | Cross sectional | 2009 | Shanghai | LCR (DNA) | Entertainment venues | Urine | 750 | 110 | 14.67% | Moderate | English |
| Hoek et al  (2001)37 | Cross sectional | 1998-1999 | Guangdong | LCX (DNA) | Entertainment venues | Cervical swabs | 966 | 311 | 32.19% | Moderate | English |
| Zhang et al  (2023)38 | Cross sectional | 2020 | Yunnan | PCR (DNA) | Entertainment venues | Urine | 3200 | 374 | 11.69% | Moderate | Chinese |

# Supplementary Table C.2. Studies reporting prevalence of *C. trachomatis* among MSM in China

| **Author**  **year** | **Study type** | **Dates** | **Region** | **Laboratory testing methods** | **Specimen collection**  **types** | **Participants Size** | **Number of positive persons** | **Prevalence of C. trachomatis** | **Study Quality** | **Language of publication** |
| --- | --- | --- | --- | --- | --- | --- | --- | --- | --- | --- |
| ***C. trachomatis* infection of the urethra** | | | | | | | | | | |
| Jiang et al  (2006) 39 | Cross sectional | 2003 | Jiangsu | PCR (DNA) | Urethra swab | 144 | 12 | 8.33% | Moderate | English |
| Cao et al  (2006) 40 | Cross sectional | 2003 | Jiangsu | PCR (DNA) | Urethra swab | 112 | 9 | 8.04% | High | Chinese |
| Chen et al  (2007) 41 | Cross sectional | 2006 | Guangdong | PCR (DNA) | Urethra swab | 172 | 9 | 5.23% | High | Chinese |
| Gao et al  (2010) 42 | Cross sectional | 2007-2008 | Jiangsu | PCR (DNA) | Urethra swab | 104 | 6 | 5.77% | High | Chinese |
| Chen et al  (2011) 43 | Cross sectional | 2010 | Jiangsu | PCR (DNA) | Urine | 444 | 20 | 4.50% | Moderate | Chinese |
| Zhou et al  (2013) 44 | Cross sectional | 2009-2010 | Jiangsu | PCR (DNA) | Urine | 291 | 19 | 6.53% | Moderate | Chinese |
| Zhang et al  (2014) 45 | Cross sectional | 2009 | Guangdong | PCR (DNA) | Urine | 408 | 66 | 16.18% | High | Chinese |
| Guo et al  (2014) 46 | Cross sectional | 2009-2010 | Jiangsu | PCR (DNA) | Urine | 291 | 19 | 6.53% | Moderate | Chinese |
| Lu  (2014) 47 | Cross sectional | 2012 | Jiangxi | PCR (DNA) | Urethra swab | 342 | 12 | 3.51% | High | Chinese |
| Fu et al  (2015) 48 | Cross sectional | 2009 | Jiangsu | PCR (DNA) | Urine | 413 | 27 | 6.54% | High | English |
| Zhang et al  (2017) 49 | Cross sectional | 2014-2015 | Yunnan | PCR (DNA) | Urine | 296 | 11 | 3.72% | High | English |
| Yang et al  (2018) 50 | Cross sectional | 2015-2017 | Jiangsu | PCR (DNA) | Urine | 310 | 13 | 4.19% | High | English |
| Zhou et al  (2019) 51 | Cross sectional | 2017-2018 | Jiangsu Guangdong  Hubei | PCR (DNA) | Urine | 379 | 12 | 3.17% | High | English |
| Li et al  (2019) 52 | Cross sectional | 2017-2019 | Guangdong | PCR (DNA) | Urine | 146 | 17 | 11.64% | High | Chinese |
| Zhao et al  (2020) 53 | Cross sectional | 2017-2019 | Guangdong | PCR (DNA) | Urine | 582 | 35 | 6.01% | High | Chinese |
| Yu et al  (2020) 54 | Cross sectional | 2019 | Yunnan | PCR (DNA) | Urine | 189 | 19 | 10.05% | High | Chinese |
| Yu  (2020) 55 | Cross sectional | 2018 | Guangdong | PCR (DNA) | Urine | 200 | 6 | 3.00% | High | Chinese |
| Ye et al  (2022) 56 | Cross sectional | 2020 | Liaoning | PCR (DNA) | Urine | 177 | 13 | 7.34% | High | English |
| Lin et al  (2022) 57 | Cross sectional | 2020 | Guangdong | PCR (DNA) | Urethra swab | 97 | 6 | 6.19% | High | English |
| Hu et al  (2022) 58 | Cross sectional | 2021 | Jiangsu | PCR (RNA) | Urine | 1087 | 46 | 4.23% | Moderate | English |
| Su et al  (2022) 59 | Cross sectional | 2018-2019 | Yunnan | PCR (DNA) | Urine | 933 | 124 | 13.29% | High | Chinese |
| Guo et al  (2023) 60 | Cross sectional | 2020 | Yunnan | PCR (DNA) | Urine | 1288 | 70 | 5.43% | High | Chinese |
| Ning et al  (2023) 61 | Cross sectional | 2021 | Guangdong | PCR (DNA) | Urine | 151 | 12 | 7.95% | High | Chinese |
| ***C. trachomatis* infection of the rectum** | | | | | | | | | | |
| Guo et al  (2023) 60 | Cross sectional | 2020 | Yunnan | PCR (DNA) | Swab | 1023 | 61 | 5.96% | High | Chinese |
| Gao et al  (2010) 42 | Cross sectional | 2007-2008 | Jiangsu | PCR (DNA) | Swab | 104 | 7 | 6.73% | High | Chinese |
| Yu et al  (2020) 54 | Cross sectional | 2019 | Yunnan | PCR (DNA) | Swab | 188 | 13 | 6.91 | High | Chinese |
| Ye et al  (2022) 56 | Cross sectional | 2020 | Liaoning | PCR (DNA) | Swab | 177 | 17 | 9.60% | High | English |
| Yang et al  (2018) 50 | Cross sectional | 2015-2017 | Jiangsu | PCR (DNA) | Swab | 310 | 42 | 13.55% | High | English |
| Zhang et al  (2017) 49 | Cross sectional | 2014-2015 | Yunnan | PCR (DNA) | Swab | 296 | 46 | 15.54% | High | English |
| Zhou et al  (2019) 51 | Cross sectional | 2017-2018 | Jiangsu Guangdong  Hubei | PCR (DNA) | Swab | 379 | 59 | 15.57% | High | English |
| Yu  (2020) 55 | Cross sectional | 2018 | Guangdong | PCR (DNA) | Swab | 200 | 16 | 8.00% | High | Chinese |
| Chen et al  (2007) 43 | Cross sectional | 2006 | Guangdong | PCR (DNA) | swab | 50 | 172 | 29.1% | High | Chinese |
| Lin et al  (2022) 57 | Cross sectional | 2020 | Guangdong | PCR (DNA) | swab | 81 | 18 | 22.2% | High | English |
| Ning et al  (2023) 61 | Cross sectional | 2021 | Guangdong | PCR (DNA) | swab | 125 | 29 | 23.2% | High | Chinese |
| ***C. trachomatis* infection of the oropharynx** | | | | | | | | | | |
| Guo et al  (2023) 60 | Cross sectional | 2020 | Yunnan | PCR (DNA) | Swab | 1190 | 7 | 0.59% | High | Chinese |
| Yu et al  (2020) 54 | Cross sectional | 2019 | Yunnan | PCR (DNA) | Swab | 190 | 2 | 1.05% | High | Chinese |
| Yang et al  (2018) 50 | Cross sectional | 2015-2017 | Jiangsu | PCR (DNA) | Swab | 310 | 5 | 1.61% | High | English |
| Zhang et al  (2017) 49 | Cross sectional | 2014-2015 | Yunnan | PCR (DNA) | Swab | 296 | 7 | 2.36% | High | English |
| Zhou et al  (2019) 51 | Cross sectional | 2017-2018 | Jiangsu Guangdong  Hubei | PCR (DNA) | Swab | 379 | 6 | 1.58% | High | English |
| Yu  (2020) 55 | Cross sectional | 2018 | Guangdong | PCR (DNA) | Swab | 200 | 1 | 0.50% | High | Chinese |

# Supplementary Table D. AHRQ cross-sectional quality evaluatio

Question 1) Define the source of information (survey, record review)

Question 2) List inclusion and exclusion criteria for exposed and unexposed subjects (cases and controls) or refer to previous publications.

Question 3) Indicate time-period used for identifying patients.

Question 4) Indicate whether or not subjects were consecutive if not population based.

Question 5) Indicate if evaluators of subjective components of study were masked to other aspects of the status of the participants.

Question 6) Describe any assessments undertaken for quality assurance purposes (e.g., test/retest of primary outcome measurements)

Question 7) Explain any patient exclusions from analysis.

Question 8) Describe how confounding was assessed and/or controlled.

Question 9) If applicable, explain how missing data were handled in the analysis.

Question 10) Summarize patient response rates and completeness of data collection.

Question 11) Clarify what follow-up, if any, was expected and the percentage of patients for which incomplete data or follow-up was obtained.

| **Author, Year** | **Q1** | **Q2** | **Q3** | **Q4** | **Q5** | **Q6** | **Q7** | **Q8** | **Q9** | **Q10** | **Q11** | **Total**  **score** | **Quality level** |
| --- | --- | --- | --- | --- | --- | --- | --- | --- | --- | --- | --- | --- | --- |
| FSWs | | | | | | | | | | | | | |
| Peng et al  (2004) | YES | YES | YES | NO | NO | YES | YES | NO | YES | YES | YES | 8 | High |
| Yang et al  (2004) | YES | YES | YES | NO | NO | YES | YES | NO | YES | YES | YES | 8 | High |
| Yang et al  (2006) | YES | NO | YES | NO | NO | YES | NO | NO | NO | YES | YES | 5 | Moderate |
| Zhu et al  (2006) | YES | YES | YES | NO | NO | YES | NO | YES | NO | YES | YES | 7 | Moderate |
| Gao  (2007) | YES | YES | YES | NO | NO | YES | NO | YES | NO | YES | YES | 7 | Moderate |
| Wang et al  (2007) | YES | YES | YES | NO | NO | YES | YES | NO | YES | YES | YES | 8 | High |
| Zhang  (2007) | YES | YES | YES | NO | NO | YES | NO | YES | NO | YES | YES | 7 | Moderate |
| Xu et al  (2008) | YES | YES | YES | NO | NO | YES | YES | NO | YES | YES | YES | 8 | High |
| Wang et al  (2008) | YES | YES | YES | NO | NO | YES | YES | YES | YES | YES | YES | 9 | High |
| Jin  (2010) | YES | YES | YES | NO | NO | NO | YES | YES | YES | YES | YES | 8 | High |
| Zhong et al  (2010) | YES | NO | YES | NO | NO | YES | YES | YES | YES | YES | YES | 8 | High |
| Jin et al  (2011) | YES | YES | YES | NO | NO | YES | NO | YES | YES | YES | YES | 8 | High |
| Li et al  (2011) | YES | YES | YES | NO | NO | NO | NO | NO | YES | YES | YES | 6 | Moderate |
| Feng  (2012) | YES | YES | YES | NO | NO | YES | YES | NO | YES | YES | YES | 8 | High |
| Zhang et al  (2012) | YES | YES | YES | NO | YES | YES | NO | YES | YES | YES | YES | 9 | High |
| Chen et al  (2013) | YES | YES | YES | NO | NO | YES | YES | YES | YES | YES | YES | 9 | High |
| Han et al  (2013) | YES | YES | YES | NO | YES | NO | NO | YES | YES | YES | YES | 8 | High |
| Tang et al  (2014) | YES | YES | YES | NO | NO | YES | YES | YES | YES | YES | YES | 9 | High |
| Han et al  (2014) | YES | YES | YES | NO | NO | YES | YES | YES | YES | YES | YES | 9 | High |
| Xu et al  (2014) | YES | NO | NO | NO | NO | YES | YES | YES | YES | YES | YES | 7 | Moderate |
| Luo et al  (2015) | YES | YES | YES | NO | NO | YES | YES | NO | YES | YES | YES | 8 | High |
| Li et al  (2015) | YES | YES | YES | NO | NO | YES | YES | YES | YES | YES | YES | 9 | High |
| Fan et al  (2016) | YES | YES | YES | NO | NO | YES | NO | NO | YES | YES | YES | 7 | Moderate |
| Guo et al  (2017) | YES | YES | YES | NO | NO | YES | NO | YES | YES | YES | YES | 8 | High |
| Li et al  (2017) | YES | YES | YES | NO | NO | YES | NO | NO | YES | YES | YES | 7 | Moderate |
| Yang et al  (2020) | YES | YES | YES | NO | NO | YES | YES | NO | YES | YES | YES | 8 | High |
| Dong et al  (2021) | YES | YES | YES | NO | NO | YES | YES | YES | YES | YES | YES | 9 | High |
| Chen et al  (2022) | YES | NO | YES | NO | NO | YES | YES | NO | YES | YES | YES | 7 | Moderate |
| Ma et al  (2022) | YES | NO | YES | NO | NO | YES | YES | YES | YES | YES | YES | 8 | High |
| Qi et al  (2022) | YES | YES | YES | NO | NO | YES | NO | NO | YES | YES | YES | 7 | Moderate |
| Zha et al (2011) | YES | NO | YES | NO | NO | YES | NO | YES | YES | NO | YES | 6 | Moderate |
| Li et al  (2009) | YES | YES | YES | NO | NO | YES | NO | YES | YES | YES | YES | 8 | High |
| Chen et al  (2005) | YES | NO | YES | NO | YES | YES | NO | YES | YES | NO | YES | 7 | Moderate |
| Shi et al  (2022) | YES | NO | YES | NO | YES | YES | NO | YES | YES | YES | YES | 8 | High |
| Wang et al  (2009) | YES | YES | YES | NO | NO | YES | YES | YES | YES | YES | YES | 9 | High |
| Remis et al  (2014) | YES | NO | YES | NO | YES | YES | NO | YES | YES | NO | YES | 7 | Moderate |
| Hoek et al  (2001) | YES | NO | YES | NO | YES | YES | NO | YES | YES | NO | YES | 7 | Moderate |
| Zhang et al  (2023) | YES | YES | YES | NO | NO | YES | NO | YES | YES | NO | YES | 7 | Moderate |
| MSM | | | | | | | | | | | | | |
| Jiang et al  (2006) | YES | YES | YES | NO | NO | YES | NO | NO | YES | YES | YES | 7 | Moderate |
| Cao et al  (2006) | YES | NO | YES | NO | YES | NO | YES | YES | YES | YES | YES | 8 | High |
| Chen et al  (2007) | YES | NO | YES | NO | YES | YES | YES | YES | YES | YES | YES | 9 | High |
| Gao et al  (2010) | YES | YES | YES | NO | YES | YES | YES | YES | YES | YES | YES | 10 | High |
| Chen et al  (2011) | YES | NO | NO | NO | NO | YES | YES | NO | YES | YES | YES | 6 | Moderate |
| Zhou et al  (2013) | YES | YES | YES | NO | NO | YES | NO | NO | NO | YES | YES | 6 | Moderate |
| Guo et al  (2013) | YES | YES | YES | NO | NO | YES | NO | NO | NO | YES | YES | 6 | Moderate |
| Lu et al  (2014) | YES | YES | YES | NO | YES | YES | YES | YES | YES | YES | YES | 10 | High |
| Zhang et al  (2014) | YES | YES | YES | NO | NO | YES | YES | NO | YES | YES | YES | 8 | High |
| Fu et al  (2015) | YES | YES | YES | NO | NO | YES | YES | NO | YES | YES | YES | 8 | High |
| Zhang et al  (2017) | YES | YES | YES | NO | NO | YES | YES | NO | YES | YES | YES | 8 | High |
| Yang et al  (2018) | YES | YES | YES | NO | YES | YES | YES | NO | NO | YES | YES | 8 | High |
| Zhou et al  (2019) | YES | YES | YES | NO | NO | NO | YES | YES | YES | YES | YES | 8 | High |
| Li et al  (2019) | YES | NO | YES | NO | YES | YES | YES | NO | YES | YES | YES | 8 | High |
| Zhao et al  (2020) | YES | YES | YES | NO | YES | NO | NO | YES | YES | YES | YES | 8 | High |
| Yu et al  (2020) | YES | YES | YES | NO | YES | YES | YES | NO | YES | YES | YES | 9 | High |
| Yu  (2020) | YES | YES | YES | NO | NO | YES | YES | NO | YES | YES | YES | 8 | High |
| Ye et al  (2022) | YES | YES | YES | NO | YES | YES | YES | NO | NO | YES | YES | 8 | High |
| Lin et al  (2022) | YES | YES | YES | NO | NO | NO | YES | YES | YES | YES | YES | 8 | High |
| Hu et al  (2022) | YES | YES | YES | NO | NO | YES | YES | NO | NO | YES | YES | 7 | Moderate |
| Guo et al  (2023) | YES | YES | YES | NO | YES | YES | NO | YES | YES | YES | YES | 9 | High |
| Su et al  (2022) | YES | YES | YES | NO | YES | YES | NO | YES | YES | NO | YES | 8 | High |
| Ning et al  (2023) | YES | YES | YES | NO | YES | YES | NO | YES | YES | NO | YES | 8 | High |

# Supplementary Figure A. and B. Forest plots presenting outcomes of the pooled prevalence among FSWs (Fig. A) and MSM (Fig. B) in China


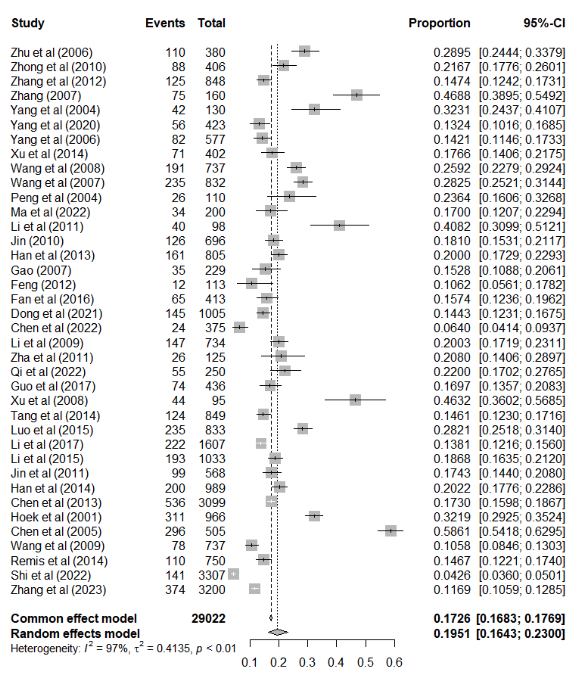


## Fig.A.1. The pooled prevalence of *C. trachomatis* in FSWs


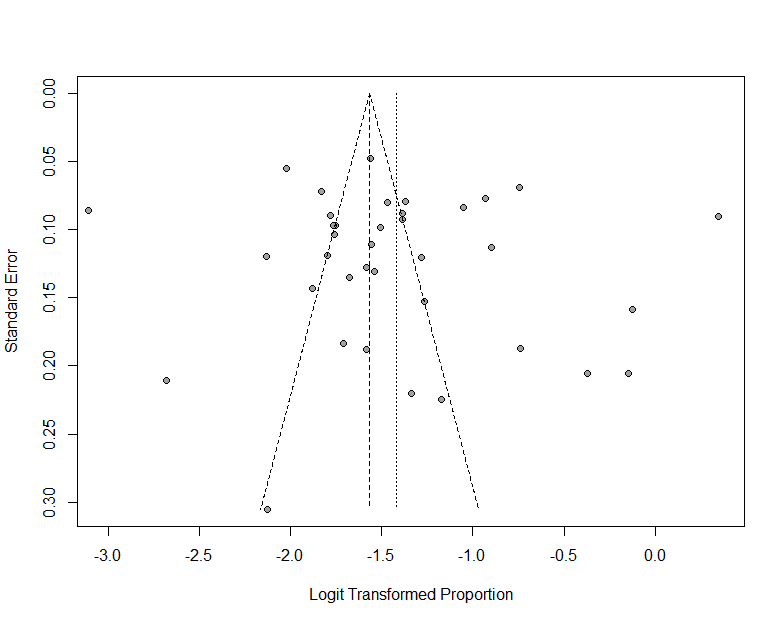


## Fig.A.2. Funnel Chart in FSWs


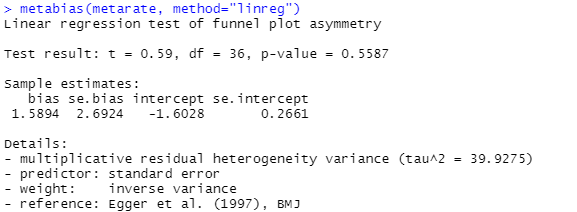


## Fig.A.3. Egger’s test in FSWs


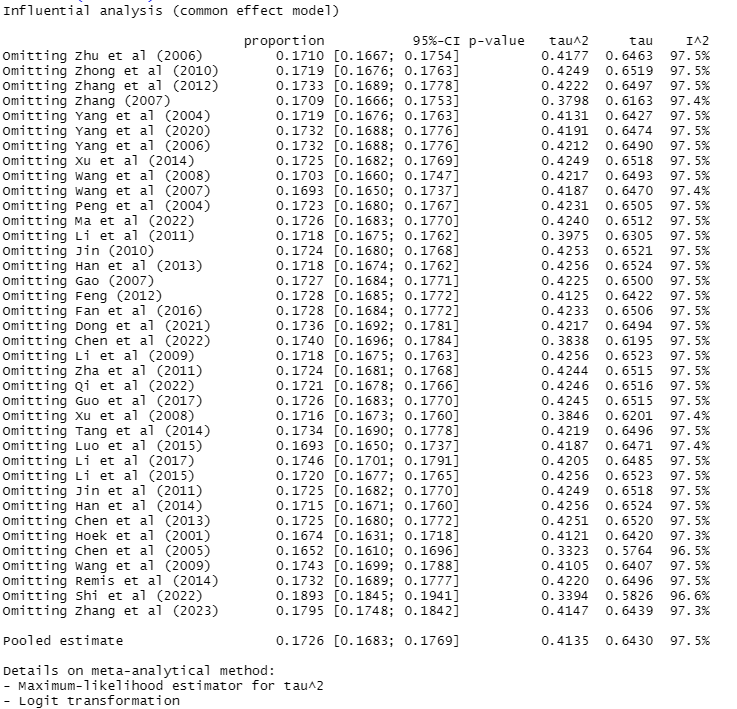


## Fig.A.4. Sensitivity Analysis in FSWs


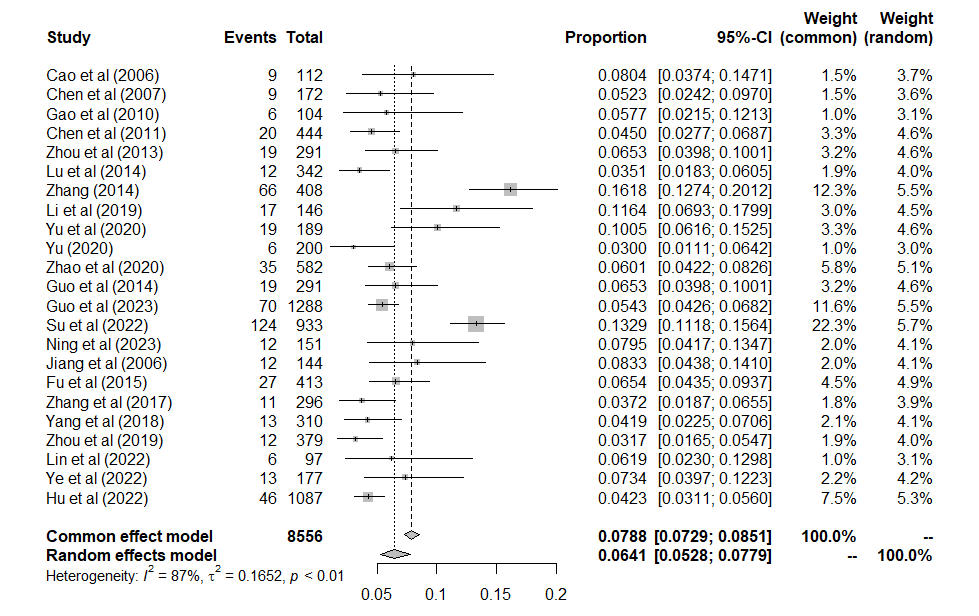


## Fig.B.1. The pooled prevalence of *C. trachomatis* in MSM


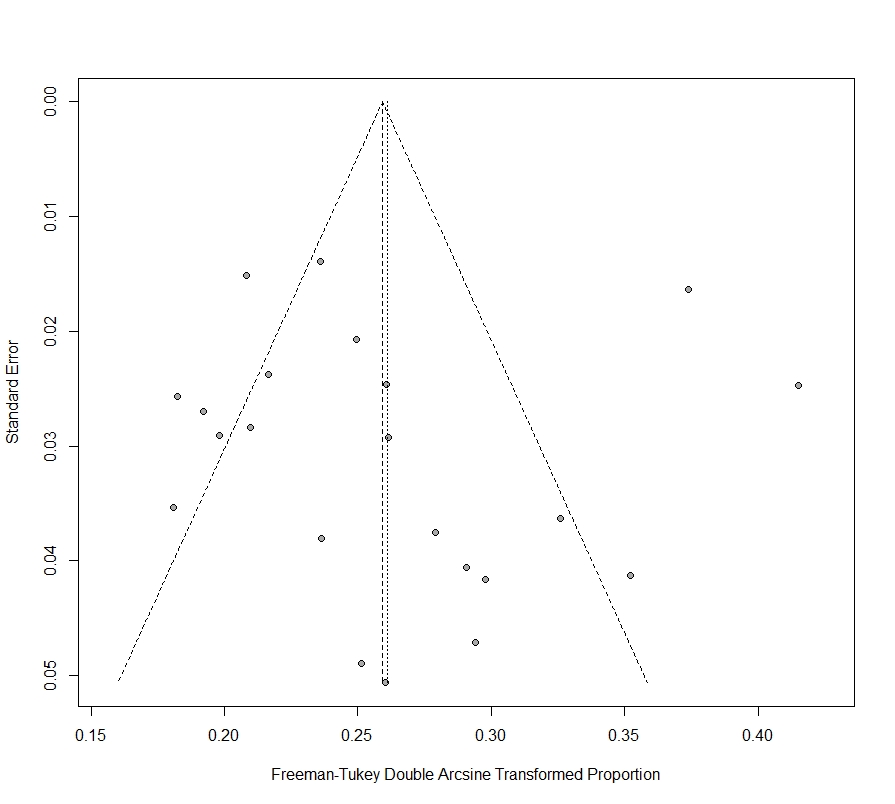


## Fig.B.2. Funnel Chart in MSM


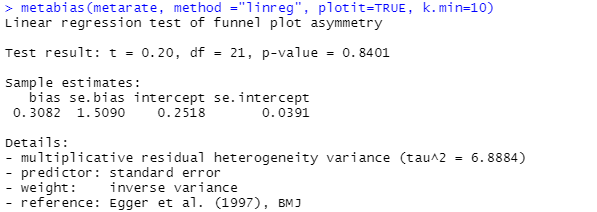


## Fig.B.3. Egger’s Test in MSM


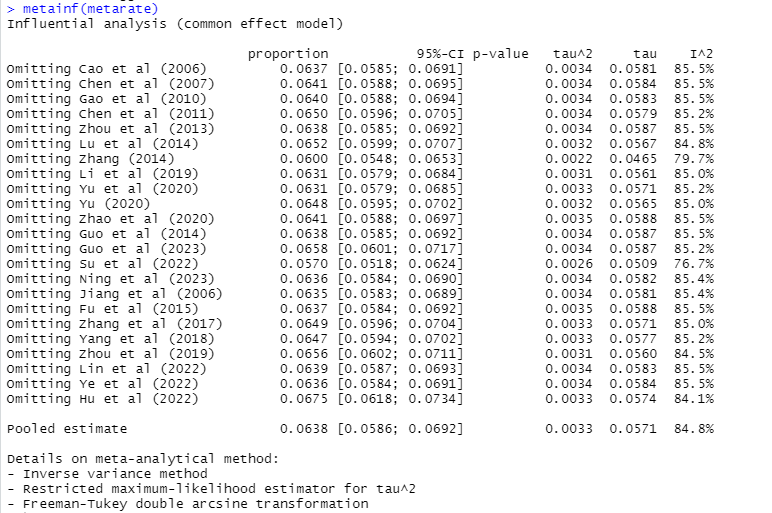


## Fig.B.4. Sensitivity Analysis in MSM

# Supplementary Figure C. and D. Forest plots presenting outcomes of the pooled prevalence among FSWs and MSM in China by Subgroup analysis


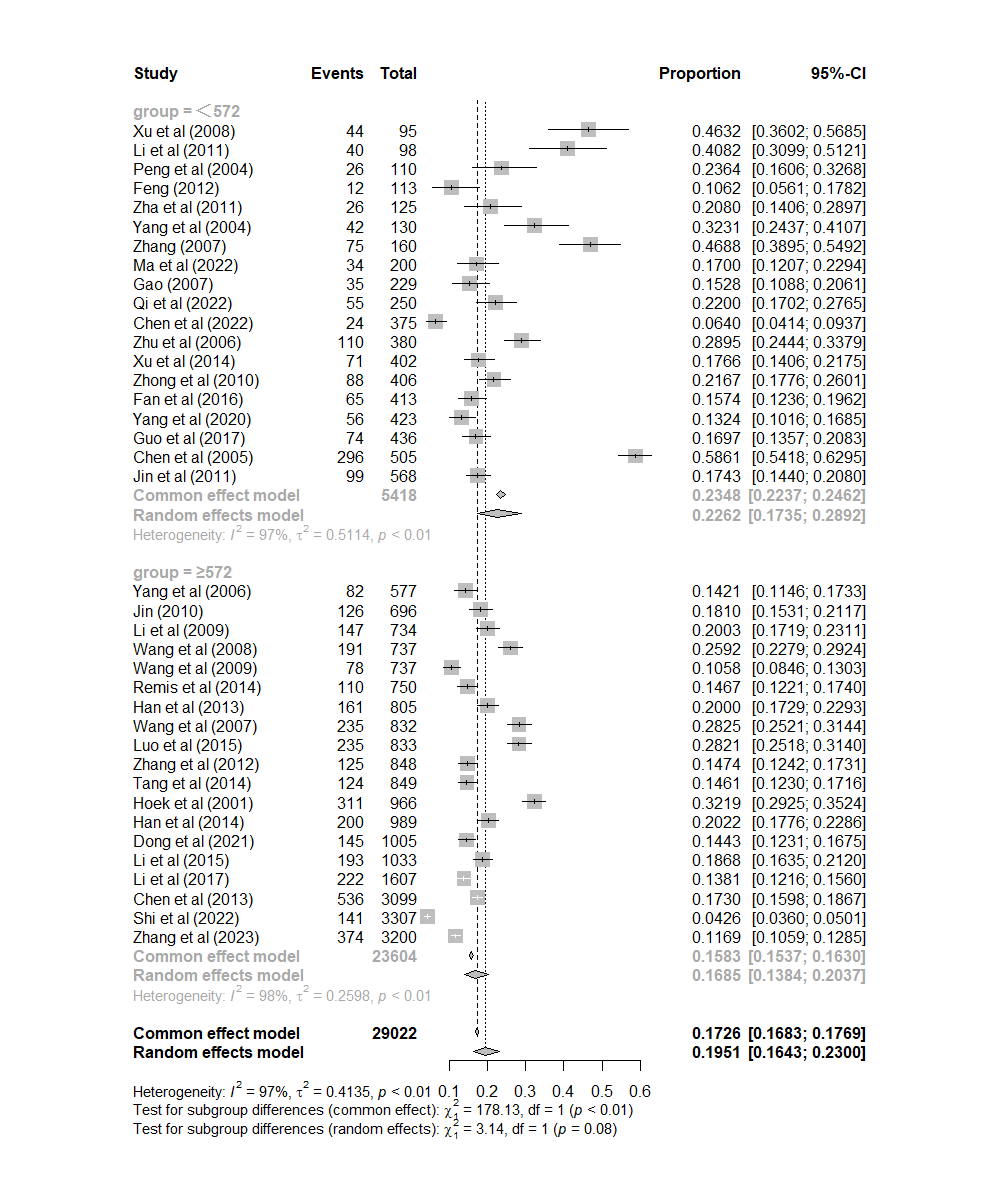


## Fig.C.1. The pooled prevalence among FSWs in the subgroup analysis of sample size


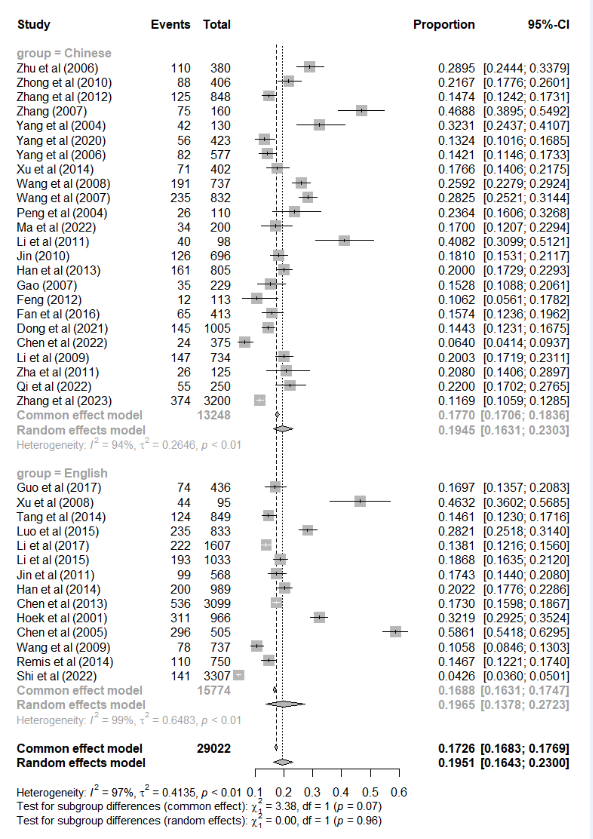


## Fig.C.2. The pooled prevalence among FSWs in the subgroup analysis of publication language

##
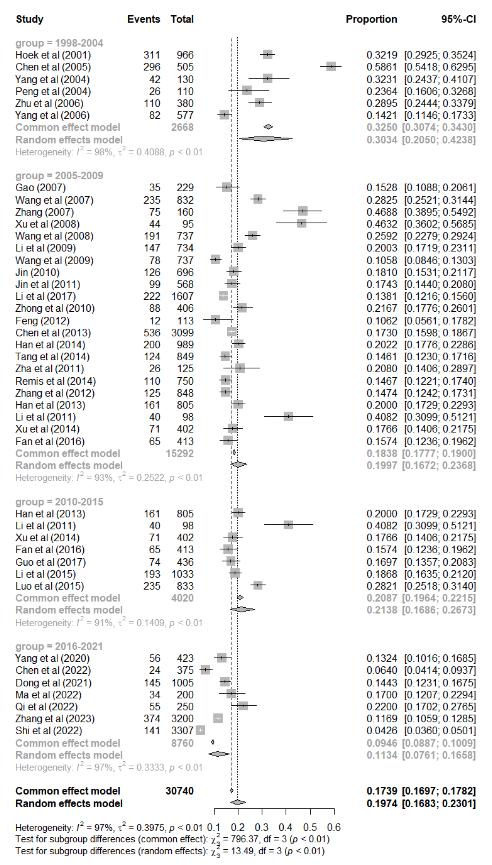


## Fig.C.3. The pooled prevalence among FSWs in the subgroup analysis of data collection period

**
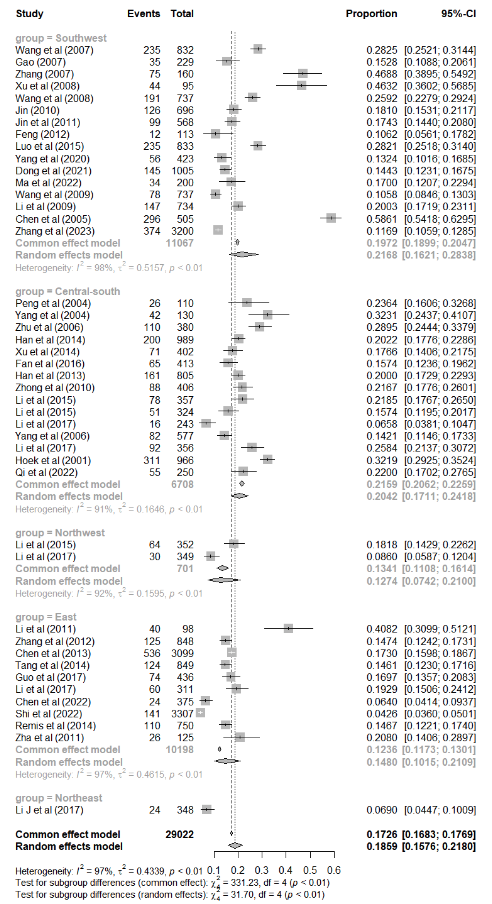
**

## Fig.C.4. The pooled prevalence among FSWs in the subgroup analysis of study region


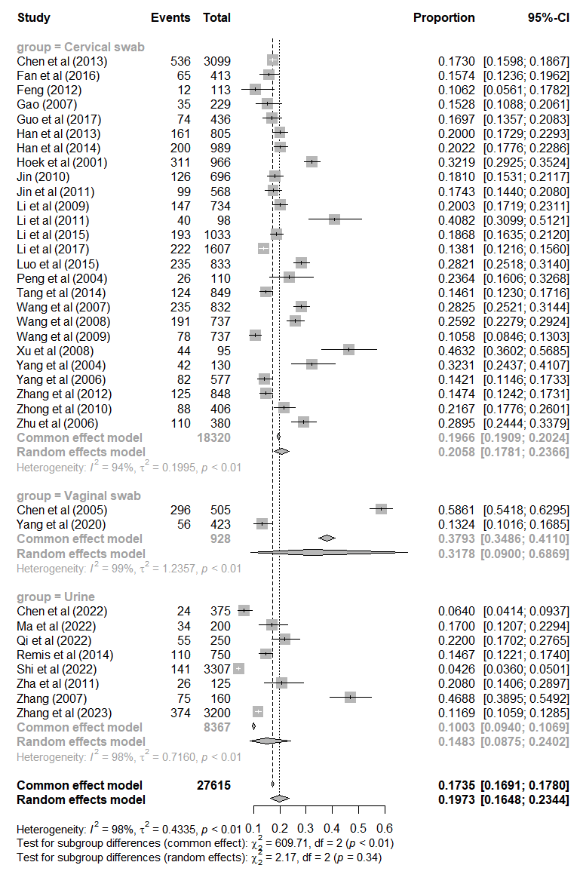


## Fig.C.5. The pooled prevalence among FSWs in the subgroup analysis of study specimen collection types


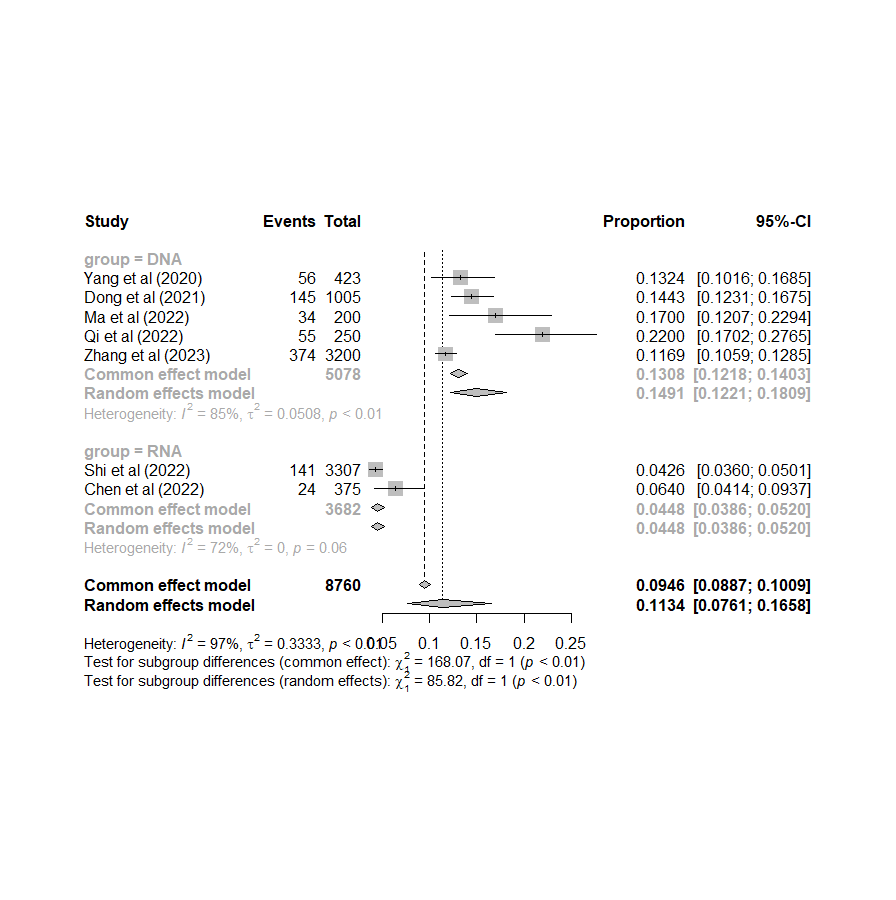


## Fig.C.6. The pooled prevalence among FSWs in the subgroup analysis of molecular diagnosis methods


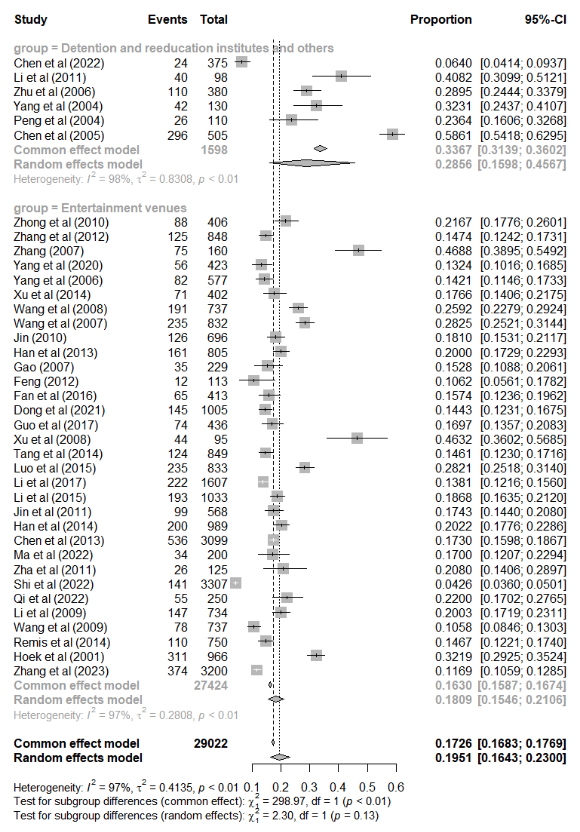


## Fig.C.7. The pooled prevalence among FSWs in the subgroup analysis of study recruitment sites


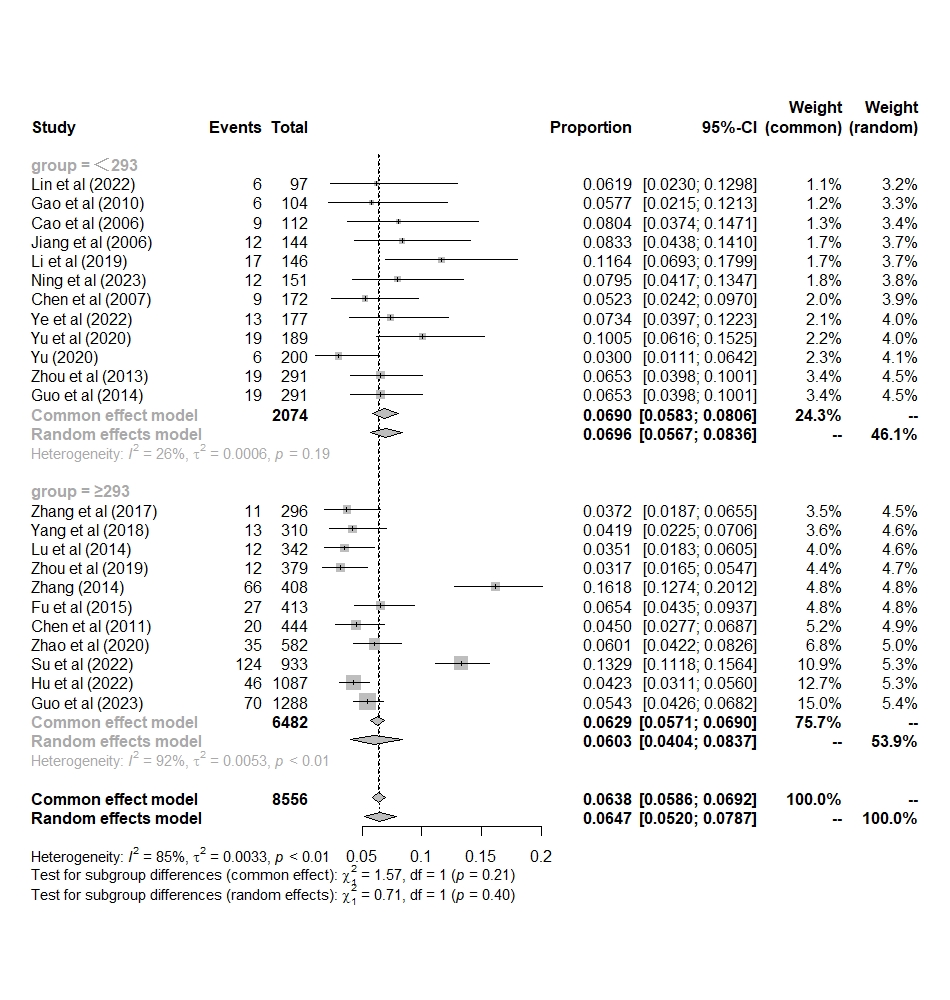


## Fig.D.1. The pooled prevalence among MSM in the subgroup analysis of sample size


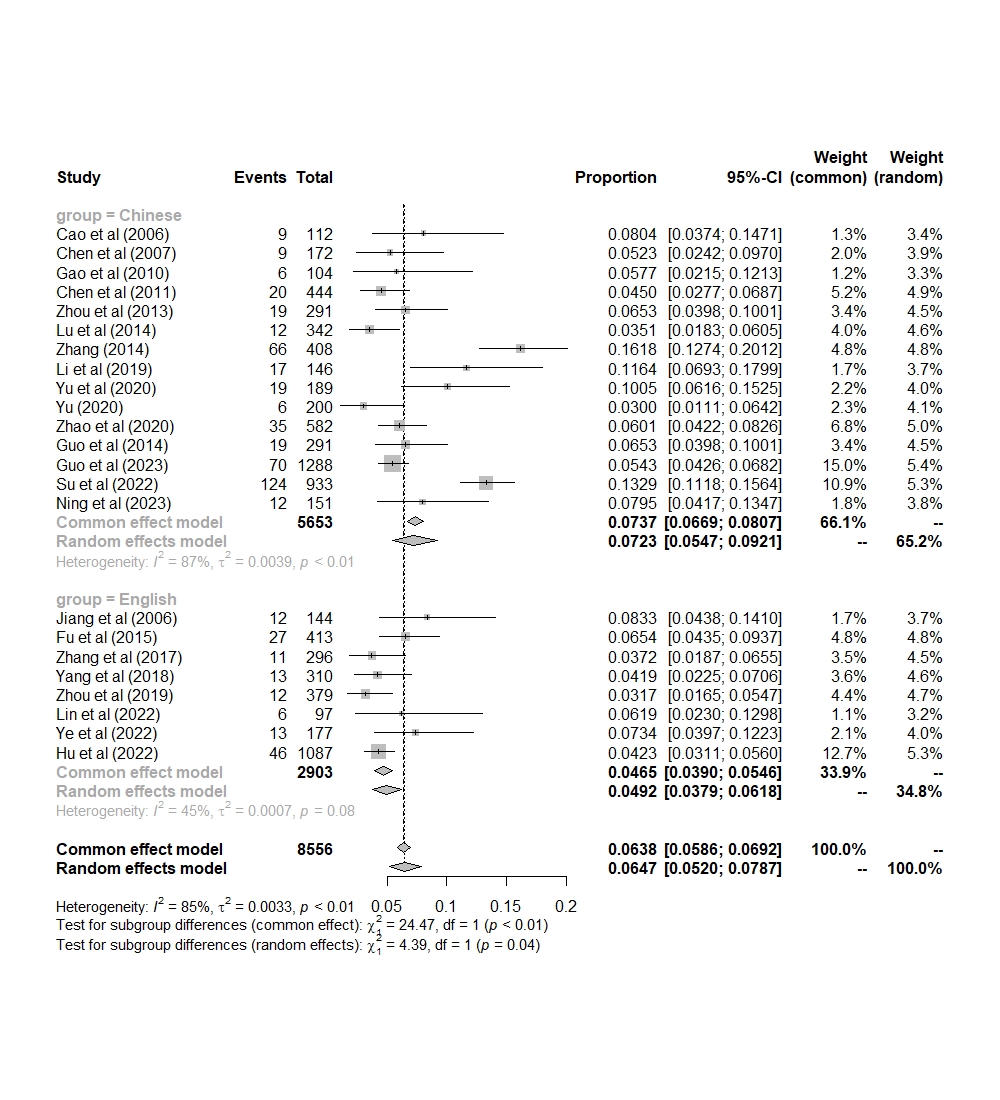


## Fig.D.2. The pooled prevalence among MSM in the subgroup analysis of publication language


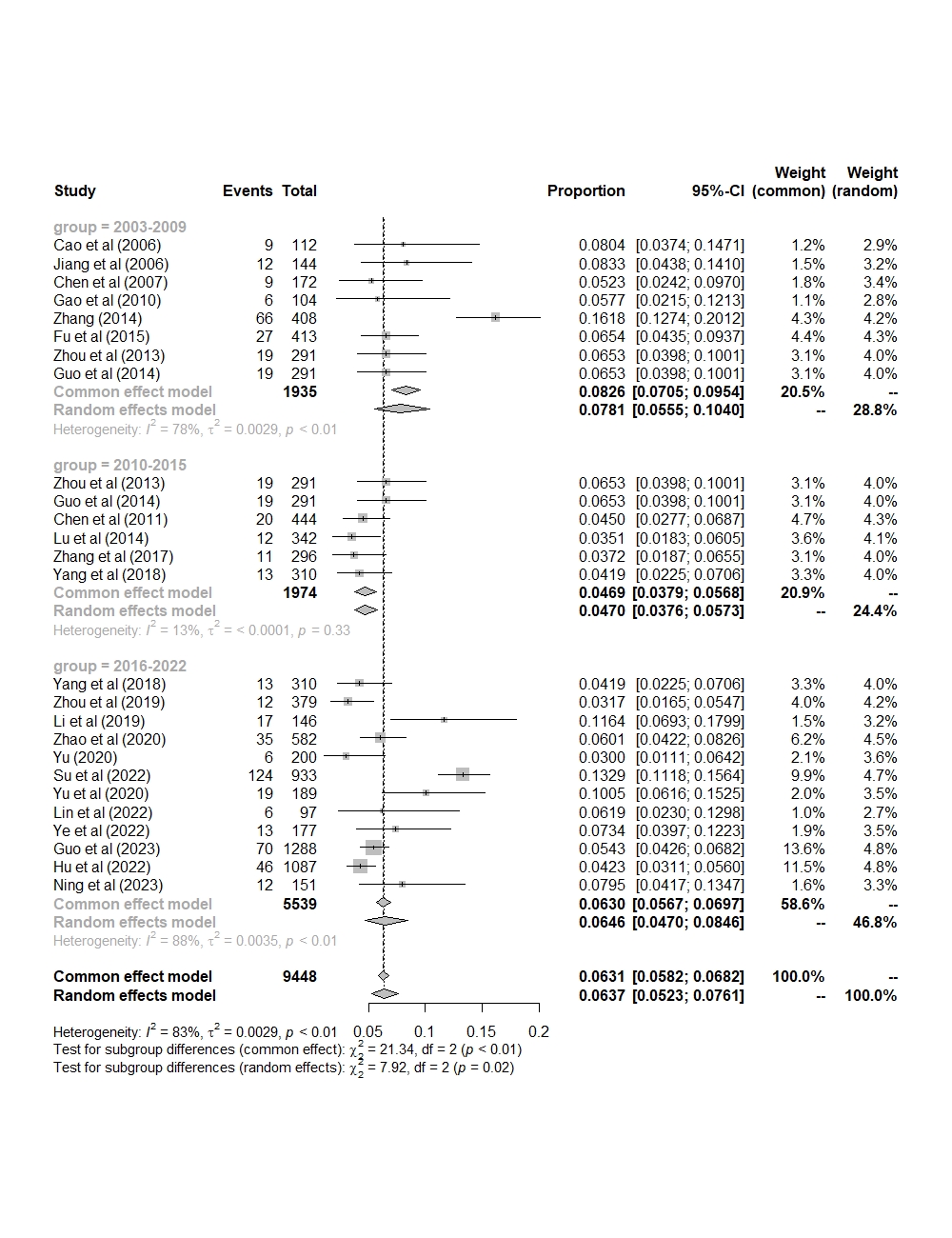


## Fig.D.3. The pooled prevalence among MSM in the subgroup analysis of data collection period


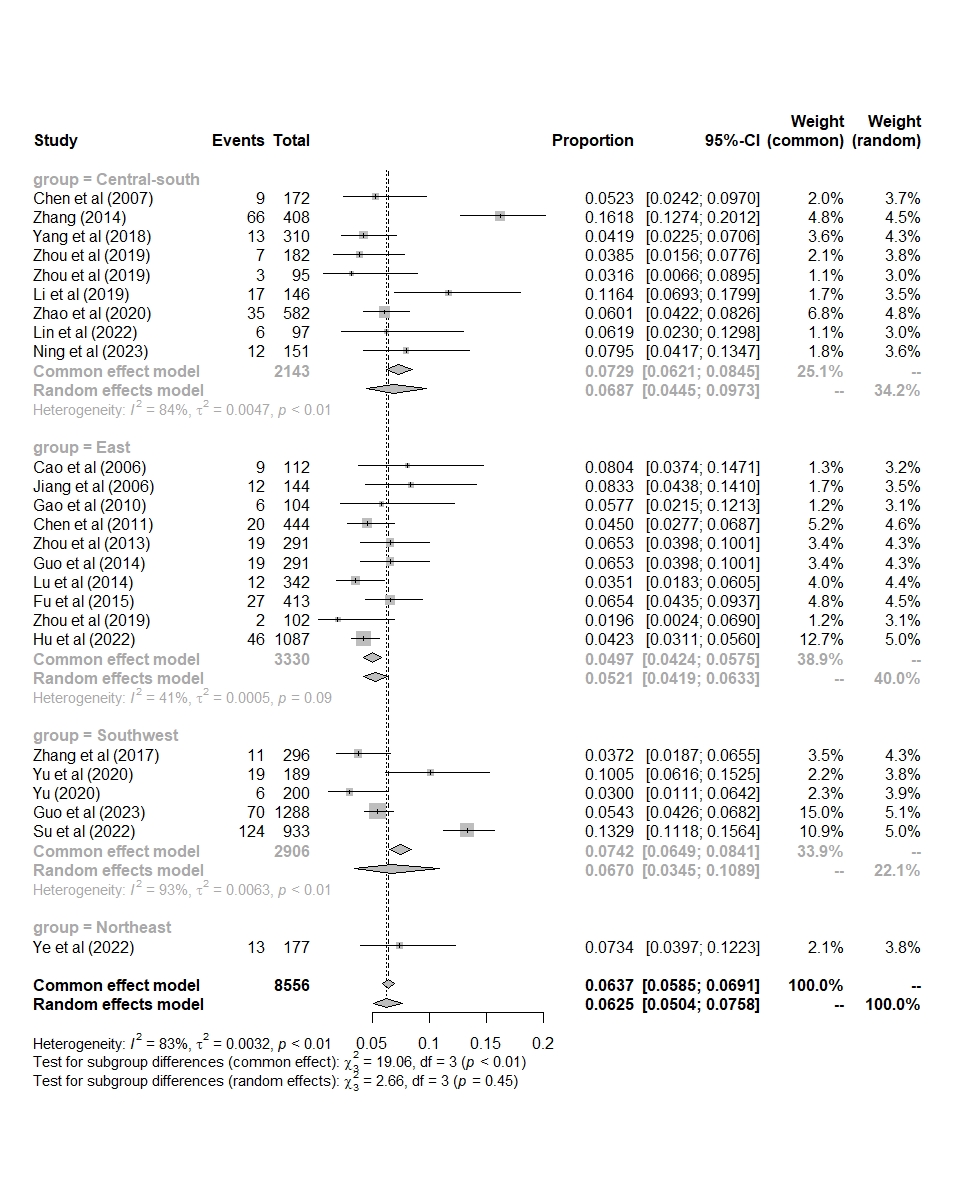


## Fig.D.4. The pooled prevalence among MSM in the subgroup analysis of study region


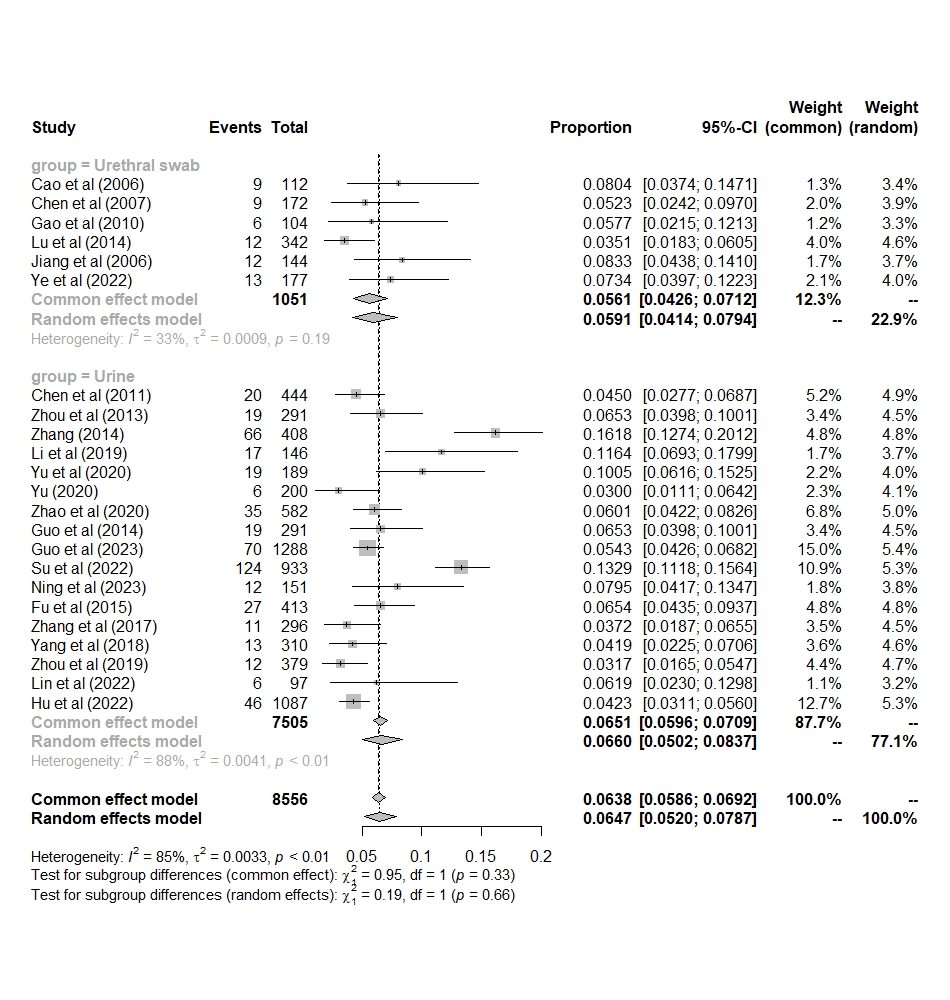


## Fig.D.5. The pooled prevalence among MSM in the subgroup analysis of specimen collection types


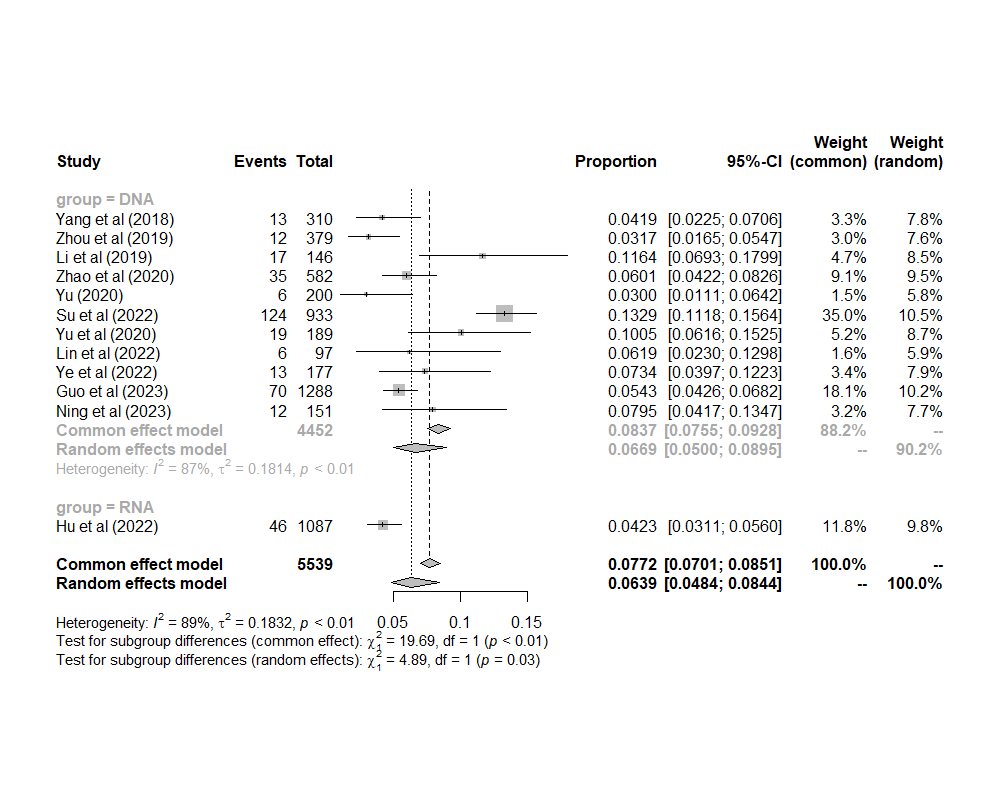


## Fig.D.6. The pooled prevalence among MSM in the subgroup analysis of molecular diagnosis methods


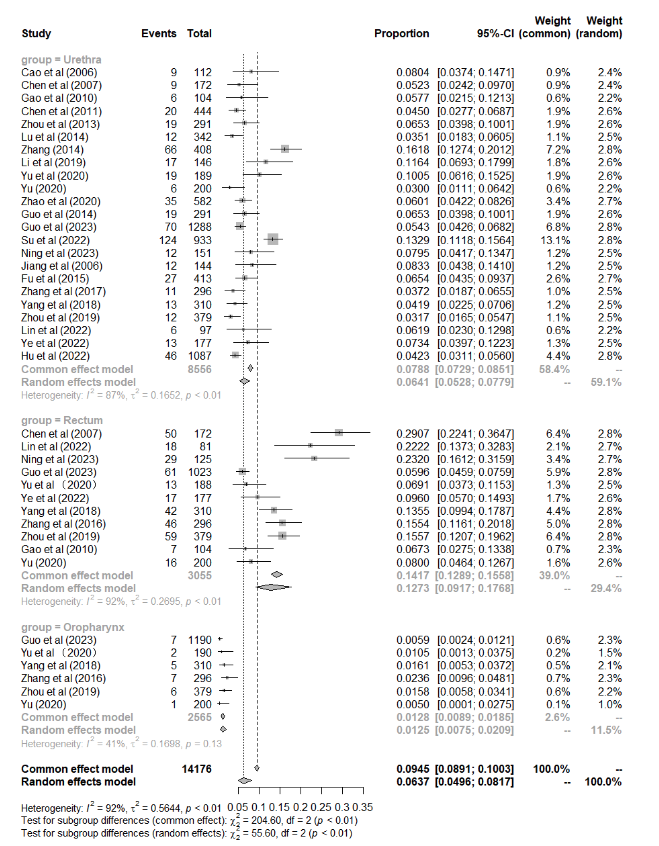


## Fig.D.7. The pooled prevalence among MSM in the subgroup analysis of specimen collection anatomical sites

# Supplementary Table E. Sensitivity analyses with restricted studies in specific conditions

|  | **Number of studies** | **Prevalence, % (95% CI)** | ***I*^2^ (%)** | ***p*-value** |
| --- | --- | --- | --- | --- |
| All studies (FSWs) | 38 | 19.5 (16.4-23.0) | 97.0 | <0.01 |
| Excluding studies with extreme C. trachomatis prevalence | 37 | 18.7 (16.0-21.8) | 97.0 | <0.01 |
| All studies (MSM) | 23 | 6.4 (5.3-7.8) | 87.0 | <0.01 |
| Excluding studies with extreme C. trachomatis prevalence | 22 | 6.1 (5.1-7.3) | 82.0 | <0.01 |

# References

1. Peng SQ, Hong FC, Li YS, et al. Investigation of Chlamydia trachomatis infection in 110 female sex workers. Modern preventive medicine. 2004(03):413-4. https://doi.org/10.3969/j.issn.1003-8507.2004.03.049.

2. Yang F, Li YS, Zhou H, et al. Detection of sexually transmitted infections among detained female commercial sex workers by fluorescent quantitative PCR assay in Shenzhen. 2004(04):334-5. https://doi.org/10.3969/j.issn.1009-1157.2004.04.011.

3. Yang BF, Xu J, Wang X, et al. Investigation on high risk behavior and chlamydia trachomatis infection in prostitutes. Journal of Public Health and Preventive Medicine. 2006(03):31-3. https://doi.org/10.3969/j.issn.1006-2483.2006.03.011.

4. Zhu DB, Pan P, Cai YM, et al. Evaluation of ClearView CT rapid test kit in testing for female genital tract Chlamydia trachomatis infection. Southern China Journal of Dermato-Venereology. 2006(04):282-4+90. https://doi.org/10.3969/j.issn.1674-8468.2006.04.007.

5. Wang HB, Wang N, Ma JG, et al. Study on the association between vaginal douching and sexually transmitted disease among Female Sex Workers in a county of Yunnan province. Chinese Journal of Epidemiology. 2007(06):558-61. https://doi.org/10.3760/j.issn:0254-6450.2007.06.011.

6. Gao HC. Epidemiology study on HIV-1 and sexually transmitted infections in a city of Yunnan Province [Master's Degree]: Southeast University; 2006. https://doi.org/[10.7666/d.y1039422](http://dx-chinadoi-cn.webvpn.njmu.edu.cn:8118/10.7666/d.y1039422).

7. Zhang GL. HIV-I and STIs prevalence and risk factors among miners and female sex workers in mining areas of Gejiu City, Honghe Prefecture. 2007.

8. Xu JJ, Wang N, Lu L, Pu Y, Zhang GL, Wong M, et al. HIV and STIs in clients and female sex workers in mining regions of Gejiu City, China. Sex Transm Dis. 2008;35(6):558-65.

9. Wang GX, Ding GW, Wang HB, et al. Two cross-sectional surveys of HIV/STD infection among female sex workers in entertainment venues in Yunnan Province. Soft science of health. 2008(05):343-5. https://doi.org/[10.3969/j.issn.1003-2800.2008.05.006](http://dx-chinadoi-cn.webvpn.njmu.edu.cn:8118/10.3969/j.issn.1003-2800.2008.05.006).

10. Jin X. Repeated cross-sectional studies of HIV/STDs among female sex workers in Kaiyuan, Yunnan Province [Master]: Chinese Union Medical College; 2009. https://kns.cnki.net/kcms/detail/detail.aspx?FileName=2010015623.nh&DbName=CMFD2010.

11. Zhong J, Lin J, Hu YM, et al. HIV/STI infection and risk behaviors among 406 commercial sex workers in Wuzhou City, Guangxi PRC. Chinese Journal of Drug Abuse Prevention and Treatment. 2010;16(06):318-21. https://doi.org/[10.3969/j.issn.1006-902X.2010.06.003](http://dx-chinadoi-cn.webvpn.njmu.edu.cn:8118/10.3969/j.issn.1006-902X.2010.06.003).

12. Jin X, Chan S, Ding G, Wang H, Xu J, Wang G, et al. Prevalence and risk behaviours for Chlamydia trachomatis and Neisseria gonorrhoeae infection among female sex workers in an HIV/AIDS high-risk area. Int J STD AIDS. 2011;22(2):80-4.

13. Li XY, Zhang YL, Zhang SP, et al. Infection of sexually transmitted diseases among 98 female sex workers. Chinese Journal of nosocomiology. 2011;21(11):2274-5. https://kns.cnki.net/kcms/detail/detail.aspx?FileName=ZHYY201111060&DbName=CJFQ2011.

14. Feng L. An Epidemiological study on the prevalence and risk factors of HIV and sexually transmitted disease among cross-border and local female sex workers in one county of Yunnan [Master]: Chinese Center for Disease Control and Prevention;2011. https://kns.cnki.net/kcms/detail/detail.aspx?FileName=1011210889.nh&DbName=CMFD2012.

15. Zhang QQ, Huan XP, Yang HT, et al. Prevalence of sexually transmitted disease and risk factors among female sex workers in Jiangsu province. Journal of Nanjing Medical University (Natural Science Edition). 2012;32(04):473-8. https://kns.cnki.net/kcms/detail/detail.aspx?FileName=NJYK201204010&DbName=CJFQ2012.

16. Han Y, Yin YP, Shi MQ, et al. Prevalence of urogenital infection with and genotype distribution of Chlamydia trachomatis among female sex workers from different entertainment venues in Wuzhou and Hezhou cities of Guangxi Zhuang Autonomous Region. Chinese Journal of Dermatology. 2013(05):313-6. https://doi.org/10. 3760/cma.j.issn.0412-4030 2013.05 004.

17. Chen XS, Yin YP, Liang GJ, Wang QQ, Jiang N, Liu Q, et al. The prevalences of Neisseria gonorrhoeae and Chlamydia trachomatis infections among female sex workers in China. BMC Public Health. 2013;13:121.

18. Han Y, Yin YP, Shi MQ, Zheng BJ, Zhong MY, Jiang N, et al. Evaluation of Abbott RealTime CT/NG assay for detection of Chlamydia trachomatis and Neisseria gonorrhoeae in cervical swabs from female sex workers in China. PLoS One. 2014;9(3):e89658.

19. Tang W, Pan J, Jiang N, Hu HY, Mahapatra T, Yin YP, et al. Correlates of chlamydia and gonorrhea infection among female sex workers: the untold story of Jiangsu, China. PLoS One. 2014;9(1):e85985.

20. Xu HW, Wang LM, Zhong N. Effect of comprehensive intervention on STD/AIDS control among female sex workers. Chinese tropical medicine. 2014;14(11):1386-7+406. https://doi.org/10.13604/j.cnki.46-1064/r.2014.11.030.

21. Li J, Jiang N, Yue X, Gong X. Vaginal douching and sexually transmitted infections among female sex workers: a cross-sectional study in three provinces in China. Int J STD AIDS. 2015;26(6):420-7.

22. Luo L, Xu JJ, Wang GX, Ding GW, Wang N, Wang HB. Vaginal douching and association with sexually transmitted infections among female sex workers in a prefecture of Yunnan Province, China. Int J STD AIDS. 2016;27(7):560-7.

23. Fan HF, Zhang ZR, Li J. A survey on STD and AIDS infections among female sex workers. Modern preventive medicine. 2016;43(08):1491-4. https://kns.cnki.net/kcms/detail/detail.aspx?FileName=XDYF201608044&DbName=CJFQ2016.

24. Guo Y, Xu X, Fu G, Huan X, Jiang N, Yin Y, et al. Risk behaviours and prevalences of HIV and sexually transmitted infections among female sex workers in various venues in Changzhou, China. Int J STD AIDS. 2017;28(11):1135-42.

25. Li J, Gong XD, Yue X, Jiang N. Dual Epidemics of Club Drug Use and Sexually Transmitted Infections among Chinese Female Sex Workers: New Challenges to STI Prevention. Biomed Res Int. 2017;2017:2093421.

26. Yang CJ, Ma YL, Chen M, et al. Epidemiological investigation of STD infection among female sex workers in Kunming, Yunnan. Chinese Journal of AIDS & STD. 2020;26(10):1110-2+5. https://doi.org/[10.13419/j.cnki.aids.2020.10.21](http://dx-chinadoi-cn.webvpn.njmu.edu.cn:8118/10.13419/j.cnki.aids.2020.10.21)

27. Ma N, Zhang WY, Yang CJ, et al. Survey on prevalence of HIV/STD infection and the related influencing factors of Chlamydia trachomatis infection among female sex workers in Qujing City, Yunnan Province. Dermatology and Venereology. 2022;44(01):1-4+18. https://doi.org/[10.3969/j.issn.1002-1310.2022.01.001](http://dx-chinadoi-cn.webvpn.njmu.edu.cn:8118/10.3969/j.issn.1002-1310.2022.01.001).

28. Chen JP, Teng Y, Li XZ, et al. Analysis of pathogen detection results of sexually transmitted diseases from 889 prostitutes and whoremongers in Hangzhou City. Chinese Journal of Health Laboratory Technology. 2022,32(05):619-621. https://kns.cnki.net/kcms/detail/detail.aspx?FileName=ZWJZ202205029&DbName=DKFX2022.

29. Dong LJ, Su XF, Chen HC, et al. Status and risk factors of Chlamydia trachomatis and Neisseria gonorrhoeae infection in genital tract of sex workers in Yunnan Province. Dermatology and Venereology. 2021;43(05):631-2+4. https://doi.org/[10.3969/j.issn.1002-1310.2021.05.007](http://dx-chinadoi-cn.webvpn.njmu.edu.cn:8118/10.3969/j.issn.1002-1310.2021.05.007).

30. Qi ZD, Zhao PZ, He SY, Zheng DY. Analysis of status and related factors of genital tract Chlamydia trachomatis infectionamong 250 female sex workers in rural areas. Journal of Diagnosis and Therapy on Dermato-venereology. 2022;29(05):457-61.

31. Zha FY, Luo Z, Gu CJ, Lu LJ, Pan QC, Liu J, et al. Survey on Sexually Transmitted lnfections and KAP among Female Entertainment Workers. Journal of Environmental and Occupational Medicine. 2011;28(10):649-51+55.

32. Li QH, Xu JJ, Wang WJ, Wang GX, Chang DF, Ding GW, et al. Survey of high risk behaviors and HlV/STD infection among FSWs from different venues of Kaiyuan City. Chinese Journal of AIDS & STD. 2009;15(02):164-6.

33. Chen XS, Yin YP, Liang GJ, Gong XD, Li HS, Poumerol G, et al. Sexually transmitted infections among female sex workers in Yunnan, China. AIDS Patient Care STDS. 2005;19(12):853-60.

34. Shi L, Luo J, Chen Y, Chen L, Hu H, Qiu T, et al. Prevalence of syphilis and chlamydia trachomatis infection among female sex workers in Jiangsu, China: Results from a multicenter cross-sectional and venue-based study. Front Public Health. 2022;10:1018724.

35. Wang H, Wang N, Bi A, Wang G, Ding G, Jia M, et al. Application of cumulative odds logistic model on risk factors analysis for sexually transmitted infections among female sex workers in Kaiyuan city, Yunnan province, China. Sex Transm Infect. 2009;85(4):290-5.

36. Remis RS, Kang L, Calzavara L, Pan Q, Liu J, Myers T, et al. Prevalence and correlates of HIV infection and sexually transmitted infections in female sex workers (FSWs) in Shanghai, China. Epidemiol Infect. 2015;143(2):258-66.

37. van den Hoek A, Yuliang F, Dukers NH, Zhiheng C, Jiangting F, Lina Z, et al. High prevalence of syphilis and other sexually transmitted diseases among sex workers in China: potential for fast spread of HIV. Aids. 2001;15(6):753-9.

38. Zhang YY, Zeng ZJ, Chen HC, Zhang XB, Yang CJ, Dai J, et al. Analysis of Chlamydia Trachomatis Genotype among Female Sex Workers in Yunnan Province. Journal of Kunming Medical University. 2023;44(02):40-4.

39. Jiang J, Cao N, Zhang J, Xia Q, Gong X, Xue H, et al. High prevalence of sexually transmitted diseases among men who have sex with men in Jiangsu Province, China. Sex Transm Dis. 2006;33(2):118-23.

40. Cao NX, Zhang JP, Xia Q, et al. Study on STD/ HIV infections among the men who have sex with men in a few cities in Jiangsu Province,China. The sixth Academic Conference of Jiangsu Sexology Association; 2006. https://kns.cnki.net/kcms/detail/detail.aspx?FileName=JSXI200606001021&DbName=CPFD2006.

41. Chen L, Feng TJ, Cai WD, et al. Study on STD/AIDS infections among men who have sex with men in Shenzhen. Chinese Journal of AIDS & STD. 2007(06):547-9. https://doi.org/[10.3969/j.issn.1672-5662.2007.06.014](http://dx-chinadoi-cn.webvpn.njmu.edu.cn:8118/10.3969/j.issn.1672-5662.2007.06.014).

42. Gao J, Zhao XP, Yang HT, et al. HIV/STDs prevalence among men who have sex with men in Suzhou of Jiangsu province. Chinese Journal of AIDS & STD. 2010;16(6):601-3. https://doi.org/10.13419/j.cnki.aids.2010.06.017.

43. Chen X, Fu GF, Xu XQ, et al. Infection status of Neisseria gonorrhoeae and Chlamydia trachomatis among men who have sex with men in Nanjing. Acta Universitatis Medicinalis Anhui. 2011;46(6):569-72. https://doi.org/10.19405/j.cnki.issn1000-1492.2011.06.018.

44. Zhou JB, Wang JT, Zhen S, et al. Effectiveness of comprehensive intervention on the prevention of AIDS among men who have sex with men. Chinese Preventive Medicine. 2013;14(06):417-21. https://doi.org/10.16506/j.1009-6639.2013.06.017.

45. Zhang DY. Epidemiological study on HPV infection and high-risk behaviors in MSM population in Shenzhen [PhD]: Peking Union Medical College; 2014. https://doi.org/[10.7666/d.Y2629335](http://dx-chinadoi-cn.webvpn.njmu.edu.cn:8118/10.7666/d.Y2629335).

46. Guo Y, Wang D, Zhou J, Chen S, Wang J, Zhen S, et al. [Effects of education level of men who have sex with men on their high risk sexual behaviors and the infection of HIV and syphilis]. Zhonghua Yu Fang Yi Xue Za Zhi. 2014;48(4):307-11.

47. Lu L, Xu D, Liu MW, et al. Study on HIV and five kinds of common STDs infection and its influencing factors among MSM in Nanchang City. Chinese Journal of Disease Control & Prevention. 2014;18(7):617-20. https://kns.cnki.net/kcms/detail/detail.aspx?FileName=JBKZ201407010&DbName=CJFQ2014.

48. Fu GF, Jiang N, Hu HY, Mahapatra T, Yin YP, Mahapatra S, et al. The epidemic of HIV, syphilis, chlamydia and gonorrhea and the correlates of sexual transmitted infections among men who have sex with men in Jiangsu, China, 2009. PLoS One. 2015;10(3):e0118863.

49. Zhang X, Jia M, Chen M, Luo H, Chen H, Luo W, et al. Prevalence and the associated risk factors of HIV, STIs and HBV among men who have sex with men in Kunming, China. Int J STD AIDS. 2017;28(11):1115-23.

50. Yang LG, Zhang XH, Zhao PZ, Chen ZY, Ke WJ, Ren XQ, et al. Gonorrhea and chlamydia prevalence in different anatomical sites among men who have sex with men: a cross-sectional study in Guangzhou, China. BMC Infect Dis. 2018;18(1):675.

51. Zhou Y, Cai YM, Li SL, Cao NX, Zhu XF, Wang F, et al. Anatomical site prevalence and genotypes of Chlamydia trachomatis infections among men who have sex with men: a multi-site study in China. BMC Infect Dis. 2019;19(1):1041.

52. Li W, Sun S, Wu QH, et al. Prevalence of Neisseria gonorrhoeae and Chlamydia trachomatis coinfection and its risk factors in Nanshan District, Shenzhen City. International Journal of Epidemiology and Infectious Disease. 2019;46(4):306-9. https://doi.org/[10.3760/cma.j.issn.1673-4149.2019.04.012](http://dx-chinadoi-cn.webvpn.njmu.edu.cn:8118/10.3760/cma.j.issn.1673-4149.2019.04.012).

53. Zhao Q, Liu LL, Yuan J, et al. Analysis of Sexually Transmitted Infections and related Factors among MSM in Shenzhen. Journal of Changzhi Medical College. 2020;34(4):258-63. https://doi.org/[10.3969/j.issn.1006-0588.2020.04.004](http://dx-chinadoi-cn.webvpn.njmu.edu.cn:8118/10.3969/j.issn.1006-0588.2020.04.004).

54. Yu LT, Zhang WY, Zhang XB, et al. Study on infection status and influencing factors of Chlamydia trachomatis and Neisseria gonorrhoeae among MSM in Yuxi and Pu'er. Chinese Journal of Public Health Management. 2020;36(5):606-10. https://doi.org/10.19568/j.cnki.23-1318.2020.05.004.

55. Yu LT. Investigation of the Chlamydia trachomatis and Neisseria gonorrhoeae infection status and influencing factors among MSM in an area of Yunnan Province [Master]. Kunming Medical University; 2020. https://doi.org/10.27202/d.cnki.gkmyc.2020.000861.

56. Ye ZH, Chen S, Liu F, Cui ST, Liu ZZ, Jiang YJ, et al. Patterns of Sexually Transmitted Co-infections and Associated Factors Among Men Who Have Sex With Men: A Cross-Sectional Study in Shenyang, China. Front Public Health. 2022;10:842644.

57. Lin XX, Meng SY, Ke WJ, Zhang XH, Wang LY, Liao YY, et al. Community engagement on-site rapid test for chlamydia and gonorrhea among men who have sex with men: a pioneering study in Guangzhou, China. BMC Public Health. 2022;22(1):1036.

58. Hu H, Chen Y, Shi L, Liu X, Xu Z, Sun L, et al. Prevalence of syphilis and chlamydia trachomatis infection among men who have sex with men in Jiangsu province, China: A cross-sectional survey. Front Public Health. 2022;10:1006254.

59. Su XF, Liu CT, Zhang XB, Guo Y, Zhang XJ, Hu T, et al. Investigationof Chlamydia trachomatis and gonococcus infections among Men who have sex with men in Yunnan province. Dermatology and Venereology. 2022;44(05):359-62.

60. Guo Y, Zhang X, Yang CJ, Ma N, Dai J, Dong LJ, et al. Prevalence and influencing factors of Chlamydia trachomatis, Neisseria gonorrhoeae andTreponema pallidum infection among men who have sex with men in Yunnanprovince,2020:a cross-sectional survey. Chinese Journal of Public Health. 2023;39(03):379-83.

61. Ning N, Cai YM, Weng RX, Wang HL, Wen LZ, Zhang CL, et al. Self-sampling acceptance and related factors of Chlamydia trachomatis among men whohave sex with men in Shenzhen. Chinese Journal of AIDS & STD. 2023;29(01):78-82.
